# Manganese Complexes Bearing Bulky DAB Ligands as Efficient Catalysts for the Solvent-Free Hydroboration of Ketones

**DOI:** 10.3390/ijms262110454

**Published:** 2025-10-28

**Authors:** Aleksandra Mermela, Agata Duch, Monika Wałęsa-Chorab, Patrycja Żak

**Affiliations:** Faculty of Chemistry, Adam Mickiewicz University in Poznań, Uniwersytetu Poznańskiego St. 8, 61-614 Poznan, Poland; aleksandra.mermela@amu.edu.pl (A.M.); agaduc@st.amu.edu.pl (A.D.); monika.walesa-chorab@amu.edu.pl (M.W.-C.)

**Keywords:** manganese complexes, 1,4-diaza-1,3-butadiene, hydroboration, boronic esters, catalysis

## Abstract

Manganese complexes of the general formula [Mn(DAB)(CO)_3_Br] featuring sterically demanding α-diimine ligands (DAB) were prepared, characterized, and found to be catalytically active in the hydroboration of ketones. The developed eco-friendly approach allowed straightforward formation of boronic esters in quantitative yields in mild and solvent-free conditions.

## 1. Introduction

**Scheme 1:**
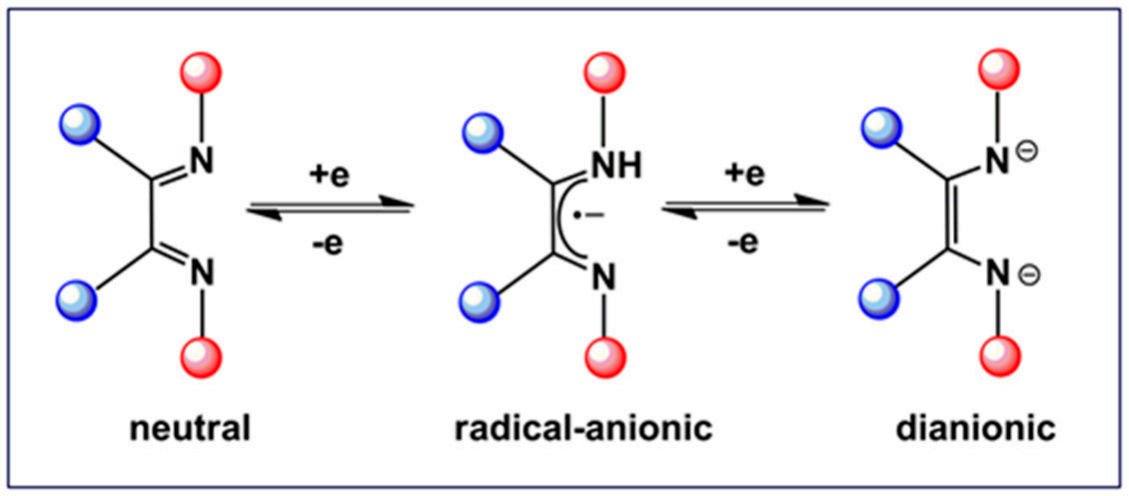
The three oxidation levels of DAB ligands.

Since the first synthesis of an α-diimine [1], molecules containing the 1,4-diaza-1,3-butadiene skeleton have attracted much interest because of both their versatile coordination behavior and the interesting properties of their metal complexes [2]. The simplest representatives of this class of compounds are 1,4-disubstituted 1,4-diaza-1,3-butadienes (α-diimines, DAB or R-DAB), which not only have a flexible N=C-C=N skeleton, but also have coordination properties as compared to the others, frequently using bidentate nitrogen donors, such as bis(imino)acenaphthene (BIAN), 2,2′-bypirydine (bipy) or phenanthroline (phen). They can coordinate to metal centers via both lone pairs of nitrogen atoms and double C=N bonds, thereby showing the ability to form a gamut of complexes in different oxidation states [3,4]. DAB ligands also have distinctive redox characteristics [5] as well as low-lying and long-lived metal-to-ligand charge transfer (MLCT) excited states [6]. They can accept one or two electrons upon reduction, thereby converting into the radical-anionic or dianionic (enediamido) form, respectively (Scheme 1).

Other advantages of α-diimines are their easily modified steric and electronic effects evoked by attaching variable substituents to the carbon and nitrogen atoms, which allows for control of the catalyst activity [4].

Over the past decades, many authors have reported effective coordination of DAB ligands to a number of the main group elements [7] and transition metals (TMs) [8,9,10,11,12,13,14]. Among them, the first-row transition metal complexes are particularly important, as they make an attractive alternative to the typically used expensive and toxic catalysts based on noble metals. Despite numerous reports on α-diimine non-noble TMs complexes, information about the synthesis of Mn-DAB systems and their application in catalysis is still scarce [5,14]. This lack is somewhat surprising, because with no doubt, manganese exhibits many desirable features. It is cheap, nontoxic and easily available, since it is widely distributed in the Earth’s crust [15]. Moreover, the catalytic applications of Mn-DAB complexes have only been investigated in a few isolated cases, with hydrosilylation reactions the most prominent examples [5]. Nonetheless, these studies clearly demonstrate the remarkable catalytic potential of α-diimine manganese complexes in the synthesis of well-defined materials of significant importance in the chemical industry.

In recent years, not only synthesis of organosilicon materials but also boron chemistry have become areas in which the application of manganese compounds has attracted increasing scientific interest. The search for new, effective and not harmful to the environment catalysts for use in the synthesis of organoboron compounds is fully justified, because these compounds can find applications in many fields, including materials chemistry, biology and medicine, as pharmaceutical agents or boron delivery agents in neutron capture therapy for cancer [16,17,18]. However, their greatest potential lies in organic synthesis [19,20,21,22], where they can serve as intermediates in the preparation of various important compounds such as alcohols. Over the past decade, several reports have demonstrated the possibility of using manganese compounds in hydroboration reactions [22,23,24,25,26,27,28,29,30,31,32,33]. Nevertheless, in most cases, the reaction conditions remained suboptimal and required further optimization in order to meet sustainability criteria. Moreover, almost all these catalysts require ligands that are often synthesized in multi-step strategies and are not stable in air.

**Figure 1:**
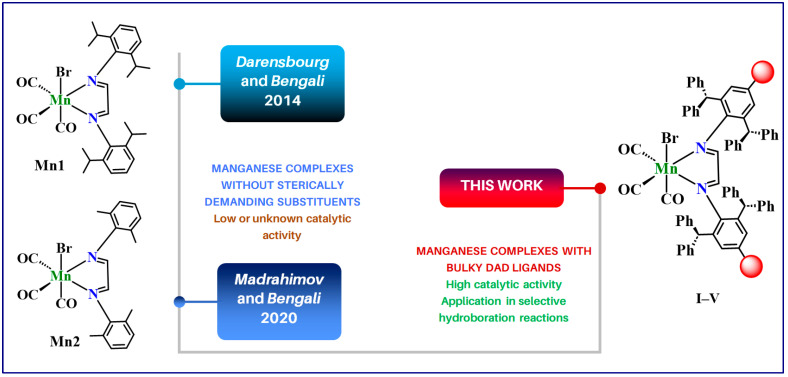
State of the art in the literature on known neutral carbonyl manganese complexes with DABs [5,14].

In this work, we report the synthesis and characterization of novel manganese complexes of the general formula [Mn(DAB)(CO)_3_Br] bearing bulky yet flexible DABs, i.e., ligands that are capable of adjusting their steric hindrance towards incoming substrates and, at the same time, are able to stabilize low-valent species (Figure 1). The properties of the synthesized complexes were also compared with those of known manganese compounds not charged with steric hindrance, previously described in the literature. The catalytical activities of novel Mn complexes were evaluated in the hydroboration process of carbonyl compounds, highlighting their efficiency under mild conditions and their potential to serve as green alternatives to traditional noble metal-based systems.

## 2. Results and Discussion

### 2.1. Synthesis and Characterization of Mn-Complexes

**Scheme 2:**
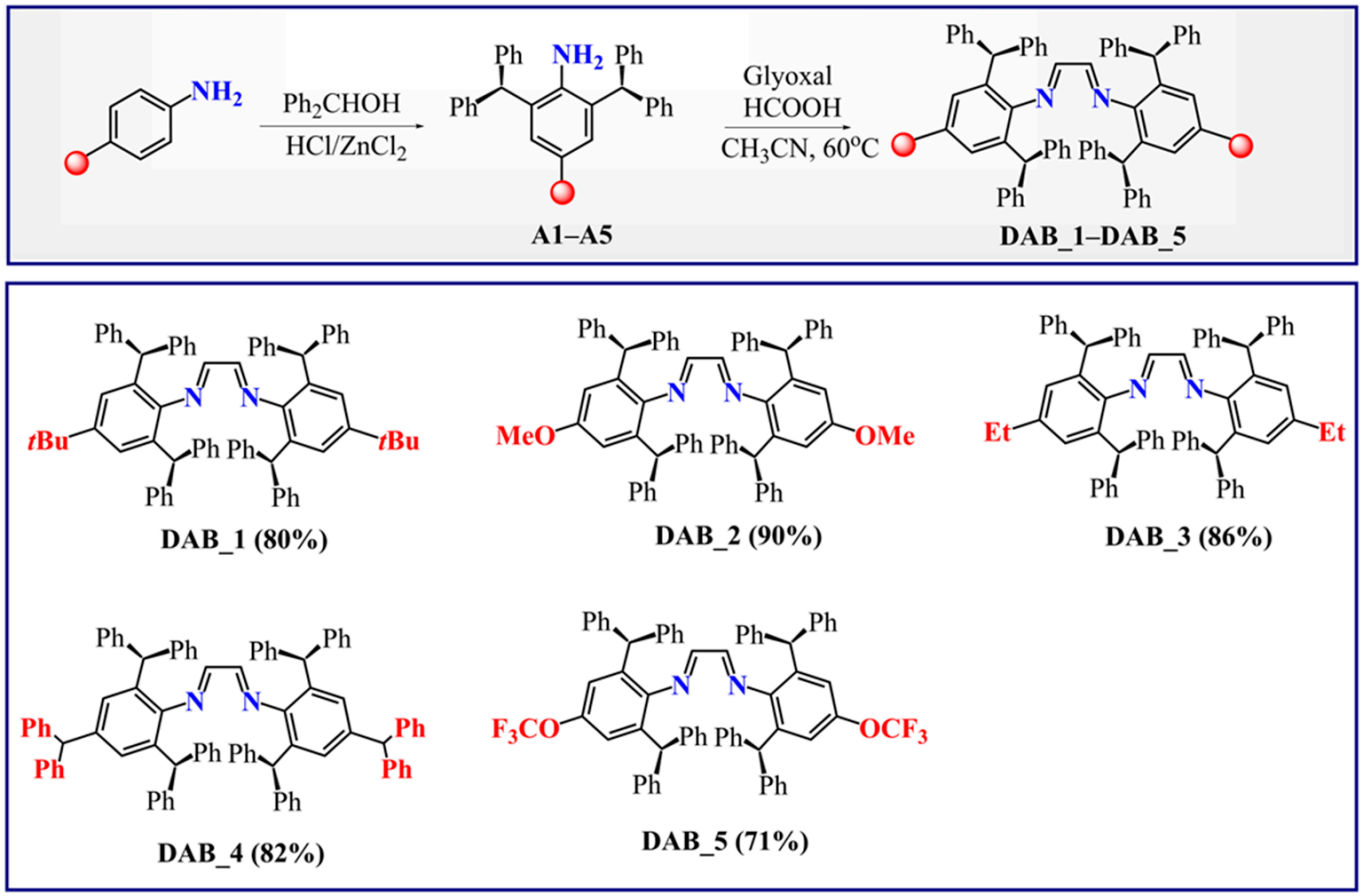
General procedure for synthesis of bulky 1,4-diaza-1,3-butadienes (DABs).

The first step of our study was the synthesis of five 1,4-diaza-1,3-butadienes containing sterically crowded substituents localized at both nitrogen atoms (DAB_1–DAB_5). Syntheses of these ligands were accomplished in two steps starting from a commercially available reagent [34,35] (Scheme 2):

The resulting DABs were isolated as yellow solids with yields in the 71–90% range, and characterized by spectroscopic and mass spectrometric methods. These compounds are air-stable, easy to handle, and can be synthesized on the gram scale. Moreover, we were able to obtain crystals of the ligands labelled DAB_3 and DAB_5 by dissolving these compounds in *n*-hexane/DCM followed by slow evaporation of the solvents. In a similar way we obtained crystals of the corresponding bulky amine (A5). The crystal structures were determined by X-ray structural analyses and confirmed the formation of the expected materials (see Supplementary Materials for details).

**Scheme 3:**
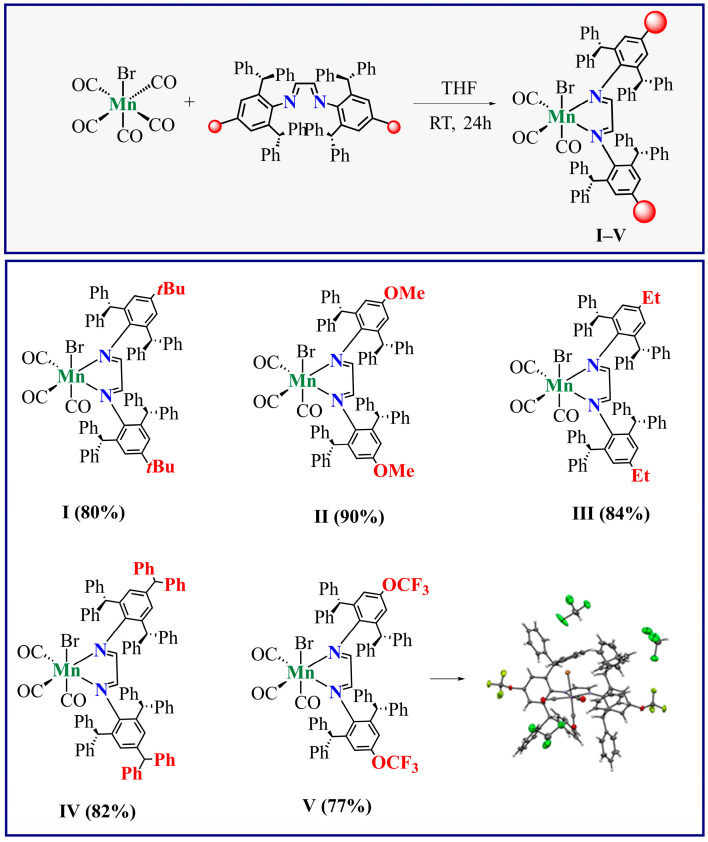
Synthesis of Mn complexes I–V**.** Perspective view of molecules of complex V.

In the next step, we applied the obtained ligands for the synthesis of new manganese complexes, according to the procedures described by Bengali’s group [14] with modifications (Scheme 3). Complexes I–V were obtained by exchanging two of the carbonyls (CO) from the commercially available bromopentacarbonylmanganese(I) [Mn(CO)_5_Br] with the appropriate DAB ligands. The syntheses were carried out under an inert gas atmosphere and in the absence of light, because of the photosensitivity of the compounds. In the same manner we obtained a known complex containing a less sterically encumbered N,N-donor ligand (Mn1) [14]. Comparison of the catalytic activity of analogous DAB-Mn systems containing non-crowded and fully-crowded substituents at the donor atoms was aimed at determining the influence of the size of the substituents on the nature of the complexes. All coordination compounds were isolated as dark violet or blue solids in yields above 77% and characterized using spectroscopic and mass spectrometric techniques. Moreover, the structure of complex V was unambiguously confirmed by XRD analysis of a single crystal obtained by dissolving this compound in dried chloroform followed by slow evaporation of the solvent (Scheme 3; see Supplementary Materials for details). The complex crystallizes in the monoclinic space group *P*2_1_/*c*. The manganese center adopts a distorted octahedral coordination geometry coordinated by two nitrogen atoms from the DAB ligand, three carbon atoms from carbonyl ligands, and one bromide anion. The Mn-N bond lengths are 2.108(3) Å and 2.055(3) Å, consistent with values reported for related manganese complexes [36,37,38]. The N-Mn-N bite angle is 77.7(1)°. Analysis of the crystal packing reveals the presence of chloroform solvent molecules, with several short intermolecular C-Cl···H and C···Cl contacts. The crystal structure of compound V shows a facial arrangement of the CO ligands, and the relative intensities of the three CO stretching bands observed in the IR spectrum are consistent with this geometry.

**Figure 2:**
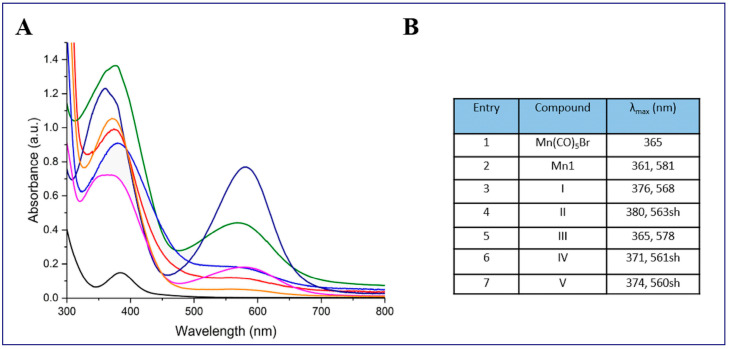
Electronic absorption spectra of [Mn(CO)_5_Br] (black) and complexes Mn1 (navy), I (olive), II (blue), III (orange), IV (magenta) and V (red), in dichloromethane at ~10^−4^ M (**A**). Numerical values of maximum absorption wavelengths are given (**B**).

In the next step, the optical properties of the manganese complexes were investigated in dichloromethane solutions at 10^−4^ M in the range of 800–300 nm. The study encompassed both the synthesized complexes with bulky (I–V) ligands and the previously reported complex with less sterically demanding ligands (Mn1), as well as a commercially available manganese precursor. The spectrum of Mn(CO)_5_Br exhibited one weak absorption band with maxima at 383 nm in dichloromethane, while the electronic absorption spectra of the complexes exhibited two transitions in the visible region (Figure 2). These bands can be attributed to metal-to-ligand charge transfer (MLCT) transitions with a contribution from bromide-to-metal charge transfer (XLCT) [39,40].

IR analysis was also performed for all complexes. The infrared spectrum of the Mn(CO)_5_Br substrate revealed characteristic signals at 2081, 2035 and 1985 cm^−1^ assigned to the stretching vibrations of carbonyl bonds, and the spectrum is consistent with those reported in the literature [41,42]. The infrared spectra of manganese complexes showed three bands assigned to carbonyl stretching vibrations ν_(C=O)_, one band to symmetric stretching at 2025 cm^−1^, and two bands attributed to asymmetric stretching at around 1963 cm^−1^ and 1911 cm^−1^ [43]. The stretching vibrations of imine C=N bonds appeared at around 1620 cm^−1^ [44]. The in-plane bending vibration of the Mn-CO group of complexes gave rise to a band at around 620 cm^−1^ and the stretching vibration of the Mn-C bond appeared at around 405 cm^−1^ [45]. Mn-Br stretching was observed at around 220 cm^−1^. The infrared spectral data on the manganese complexes are given in Table 1.

### 2.2. Electrochemistry

**Figure 3:**
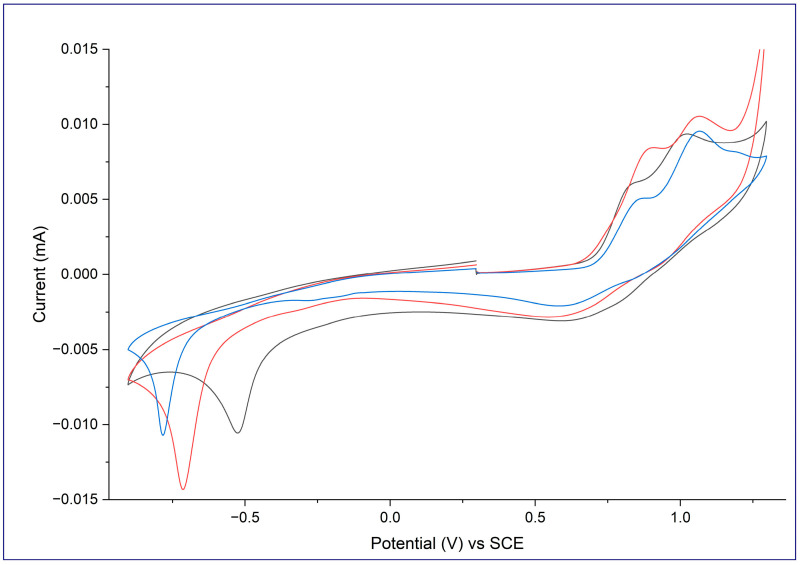
Cyclic voltammetry of complexes I (blue), III (red) and V (black) measured in anhydrous and deaerated 0.1 M solution of TBAClO_4_ in acetonitrile as a supporting electrolyte with scan speed 100 mV/s.

The electrochemical properties of Mn(I) complexes I, III and V were investigated by cyclic voltammetry (Figure 3). These three compounds were selected as representative examples, capturing the range of structural features present in the full series. The aim was to assess the redox behavior of the complexes. A 0.1 M solution of tetrabutylammonium perchlorate (TBAClO_4_) in dry acetonitrile was used as the supporting electrolyte. Measurements were performed on a three-electrode setup using a platinum working electrode (ϕ = 2 mm), an Ag/Ag^+^ reference electrode, and a platinum wire as the counter electrode. The cyclic voltammograms of all three compounds were similar and showed two oxidation waves: a quasi-reversible one, likely associated with the Mn(I)/Mn(II) redox couple [46], and an irreversible one, probably attributed to the electrochemical oxidation of the ligand [47,48]. In addition, a single irreversible reduction wave was observed, probably corresponding to the formation of a radical anion on the ligand [10]. Notably, the reduction potential shifted to more negative values, from −0.53 V for V to −0.71 V for III and −0.78 V for I, indicating that ligand reduction becomes increasingly difficult in the presence of electron-donating substituents [49,50]. The complexes were found to be unstable upon electroreduction and underwent decomposition.

### 2.3. Catalytic Investigations

The catalytic properties of the synthesized manganese complexes were tested in the hydroboration of ketones. This choice was dictated by two arguments. Firstly, the selected process permits designing compounds that may act as attractive synthons for the synthesis of molecules characterized by well-defined structures and intriguing properties [51]. Secondly, comparison of the results obtained for complexes I–V with the literature data published recently for Mn(CO)_5_Br [35] allowed for straightforward verification of the influence of the DAB ligands on the reaction course.

**Scheme 4:**
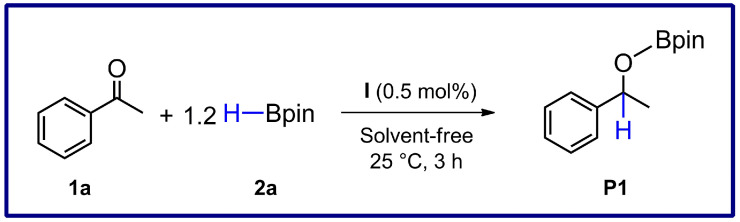
Hydroboration of acetophenone (**1a**) catalyzed by catalyst I.

We started the catalytic stage of our study with a number of tests using the same conditions as those described for Mn(CO)_5_Br in the paper by Walkowiak’s group [33]. Preliminary experiments were carried out with the use of acetophenone (**1a**) and pinacolborane (**2a**, HBpin) as model reactants. Treatment of the mixture of **1a** and **2a** in a molar ratio of 1 to 1.2 with 0.5 mol% of catalyst I resulted in complete conversion of ketone after 2 h and the formation of a single product, which was identified by GC-MS and ^1^H NMR spectroscopy as 4,4,5,5-tetramethyl-2-(1-phenylethoxy)-1,3,2-dioxaborolane (**P1**) (Scheme 4).

The outcome of this part of our study stimulated us to continue our efforts. A series of additional tests were performed to select optimal type and concentration of the catalyst and time reaction. All experiments were performed for the above model reaction. The results are summarized in Table 2.

The results gathered in Table 2 reveal that the type of manganese complex has a major impact on the outcome of the hydroboration process. Indeed, the experiments carried out in the absence of any catalyst (Table 1, Entry 15) did not afford any products, even though the reaction time was extended to 72 h. The tests performed in the presence of catalyst Mn1 containing 2,6-diisopropylphenyl groups at both nitrogen atoms led to moderate conversion of substrate **1a** (Table 1, Entries 12–14), while the presence of the complexes with bulky DAB ligands (I–V) ensured a quantitative course of the reactions (Table 1, Entries 4 and 8–11). These results clearly show that the more sterically hindered the substituents on the DAB ligand, the faster the reaction. This is also in line with literature claims that the presence of bulky ligands in the coordination sphere of transition metal complexes increases the efficiency of the catalytic processes [52,53]. Since catalysts I–V displayed similar activities in the preliminary experiments described above, we decided to continue our studies using only complex **I** (Table 1, Entry 4). Next, we optimized the concentration of the catalyst. It was observed that the amount of complex I could be reduced to 0.2 mol% without a significant loss of process effectiveness (Table 1, Entry 6). Lowering the catalyst loading to 0.1 mol% led to a reduction in the yield of product **P1,** even though the reaction time was much increased (Table 1, Entry 7). Altogether, our catalytic screening demonstrated that the best result was achieved when the reaction was carried in solvent-free conditions at 25 °C using a 1.2-fold excess of **2a** in relation to **1a** and 0.5 mol% of catalyst I (Table 1, Entry 4).

**Scheme 5:**
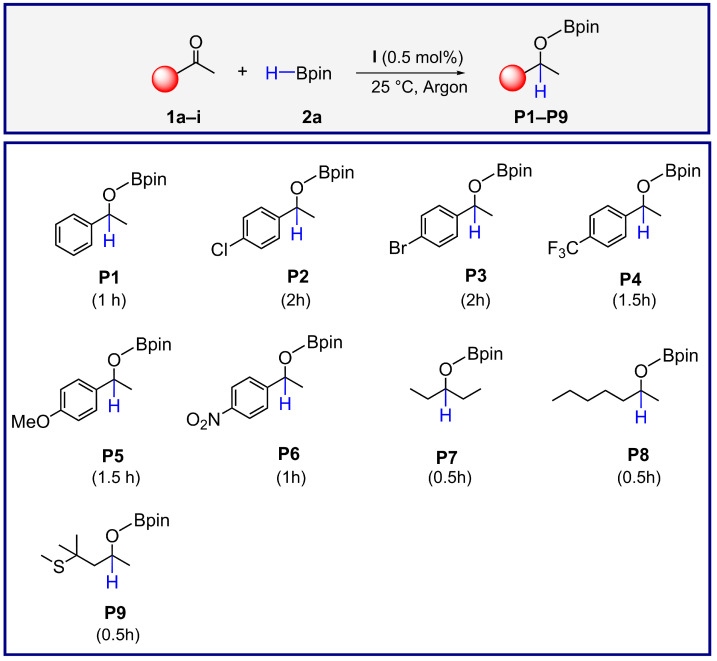
Hydroboration of ketones (**1a**–**i**) (experimental conditions: solvent-free, 25 °C, [**1a**–**i**]:[**2a**] = 1:1.2, [I] = 0.5 mol%, argon). Reaction times are given for each product under its respective structure. Quantitative yields of all products (**P1**–**P9**) were obtained.

Having established the optimized reaction conditions, the range of reagents was extended to determine the versatility of the method. Thus, we probed the reactivity of selected commercially available aromatic and aliphatic ketones (**1a**–**i**) in reactions with the most common pinacolborane (**2a**). The results are summarized in Scheme 5.

For all tested substrates, we achieved quantitative yields and complete chemoselectivity toward the formation of the expected products (**P1**–**P6**). We did not observe the formation of other products of competitive reactions for any of the reagents studied, which proves that the synthetic protocol tolerates a broad scope of moieties. It works effectively with acetophenone derivatives containing various substituents (**1a**–**f)** as well as alkyl-substituted ketones (**1g**–**i**). No significant differences in the courses of the reactions were observed, except in reaction time. In the case of aliphatic ketones (**1g**–**i**), quantitative conversion of **1g**–**i** was achieved in a shorter time compared to that needed for aromatic derivatives (**1a**–**f**). As presented in Scheme 5, all products were obtained with full atom economy in very mild conditions, which significantly simplified the final materials’ isolation stage. We decided not to isolate all products of the reactions as most of them are well known. We restricted the isolation procedure to only a few compounds, just to propose a universal method for their separation.

**Scheme 6:**
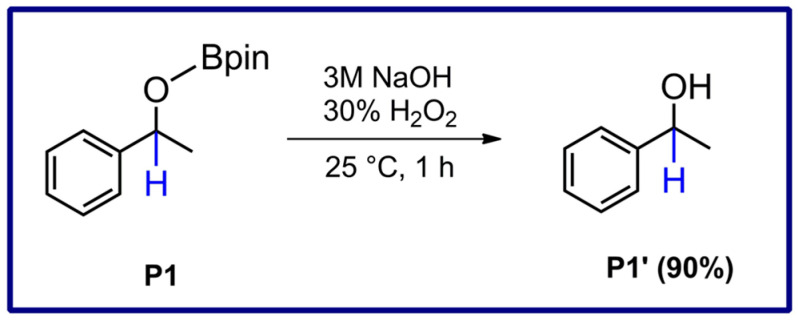
Hydrolysis of **P1**. Isolated yield is given for product **P1′** under its structure.

To illustrate the utility of the obtained boronic esters, a selected product (**P1**) was hydrolyzed leading to phenylethanol (**P1′**) (Scheme 6), which is used in perfumes thanks to its rose-like scent, as a preservative in cosmetics and soaps, and as a component in cleaning and air care materials [54]. Moreover, it also serves as a building block in pharmaceutical synthesis and has antimicrobial properties [55].

Hydrolysis of the selected boronate ester (**P1**) afforded the corresponding alcohol in high yield, which proves that the proposed synthetic protocol is a convenient method for obtaining secondary alcohols.

## 3. Materials and Methods

### 3.1. General Methods and Chemicals

All operations, except the preparation of amines and 1,4-diaza-1,3-butadienes, were carried out under dry argon using standard Schlenk techniques and vacuum gas manifold systems. The synthesis of all complexes was conducted in the dark to prevent light-induced decomposition. ^1^H NMR and ^13^C NMR spectra were recorded in CDCl_3_ or CD_2_Cl_2_ on a Varian 400 or 600 operating at 402.6, 101.2 and 600 MHz, respectively. Chemical shifts are reported in ppm with reference to the residual solvent peaks for ^1^H and ^13^C NMR. The multiplicities are reported as follows: singlet (s), doublet (d), triplet (t), quartet (q), doublet of doublets (dd), multiplet (m) and broad singlet (brs). Gas chromatography (GC) analyses were performed using an Agilent 7890B instrument (Agilent, Santa Clara, CA, USA) equipped with a thermal conductivity detector (TCD) and a DB-5 column (30 m length, 0.53 mm inner diameter). Fourier-transform infrared (FT-IR) spectra were obtained using an FT-IR INVENIO R spectrometer (Bruker, Billerica, MA, USA). Spectra were collected with a resolution of 2 cm^−1^ over the range of 4000–200 cm^−1^. UV-Vis absorption spectra were measured using a Jasco V-770 UV-Vis-NIR spectrophotometer (Jasco, Easton, MD, USA) in a spectral range from 800 nm to 300 nm. High-resolution mass spectrometry (HRMS) analyses were performed on a Maldi SYNAPT G2-S HDMS instrument (Waters, Milford, MA, USA) equipped with an electrospray ionization source, operating in the 40–8000 *m*/*z* range with a mass resolution up to 40,000. Low-resolution mass spectra (ESI-MS) were acquired using a Synapt G2-S HDMS spectrometer (Waters) featuring a quadrupole time-of-flight (Q-TOF) analyzer and an FWHM resolution of 38,000. The Capillary Voltage was set to 4.5 kV, the sampling was set to 40 and the source temperature was equal to 120 °C. Samples for mass spectrometry were prepared as solutions in methanol or methanol/chloroform mixtures. Elemental analyses were performed using a Vario EL III apparatus (Elementar Americas Inc., Ronkonkoma, NY, USA). Thin-layer chromatography (TLC) was conducted on plates coated with a 250 μm layer of silica gel and column chromatography was performed using silica gel 60 (70–230 mesh). X-ray diffraction (XRD) data were collected using a Rigaku SuperNova diffractometer (Rigaku Holdings Corporation, Tokyo, Japan) equipped with an Atlas CCD detector and monochromated CuKα radiation (λ = 1.541878 Å). Electrochemical measurements were carried out using a multichannel Bio-Logic VSP potentiostat (BioLogic, Seyssinet-Pariset, France), with a 0.1 M solution of tetrabutylammonium perchlorate (TBAClO_4_) in acetonitrile serving as the supporting electrolyte. Before use, acetonitrile was dried by passing it through neutral alumina and then stored over 3 Å molecular sieves [56]. Prior to electrochemical experiments, the solution was purged with argon for 20 min to eliminate dissolved oxygen, and an argon atmosphere was maintained throughout the measurements to prevent reintroduction of oxygen. A three-electrode setup was employed, consisting of a platinum working electrode, a non-aqueous Ag/Ag^+^ reference electrode, and a platinum wire as the counter electrode. Ferrocene was used as an external reference, and the cyclic voltammograms were calibrated vs. SCE electrode (E’_o_ = +0.38 V vs. SCE in acetinitrile with 0.1 M TBAClO_4_ as an electrolyte) [57].

All reagents, except DABs and manganese complexes, were commercially available and used as received. DAB ligands [29,30] and all catalysts [14] were prepared according to literature procedures. All solvents, except THF, were dried over CaH_2_ prior to use and stored over 4Å molecular sieves under argon. Dichloromethane was additionally passed through an alumina column and degassed by repeated freeze-pump-thaw cycles. THF was dried over sodium benzophenone ketyl and freshly distilled prior to use.

### 3.2. General Procedure for the Synthesis of Amines

A 250 mL round-bottom flask equipped with a magnetic stir bar was charged with diphenylmethanol (10 g, 5.4 × 10^−2^ mol, 2 equiv.) and 4-substituted aniline (2.7 × 10^−2^ mol, 1 equiv.). The mixture was heated in an oil bath at 60 °C until liquefaction. Subsequently, a solution of anhydrous zinc chloride (2.5 g, 1.35 × 10^−2^ mol, 0.5 equiv.) in concentrated hydrochloric acid (5 g, 2.7 × 10^−2^ mol, 1 equiv.) was added dropwise. The temperature of the oil bath was then increased to 160 °C and the reaction mixture was further stirred for 2.5 h. Upon cooling to room temperature, the glassy solid was dissolved in dichloromethane (125 mL), and the organic layer was extracted. The organic phase was washed twice with distilled water (2 × 125 mL). The combined organic extracts were concentrated under reduced pressure, and the residue was treated with 100 mL of *n*-hexane. The resulting suspension was filtered under reduced pressure through a Büchner funnel. The isolated product was dried under vacuum and characterized by spectroscopic and mass spectrometric analysis.

### 3.3. General Procedure for the Synthesis of 1,4-Diaza-1,3-Butadienes

A 500 mL single-neck round-bottom flask equipped with a magnetic stir bar was charged with amine (2.3 × 10^−2^ mol) and acetonitrile (250 mL). A 40% aqueous glyoxal solution (0.7 g, 1.15 × 10^−2^ mol) and formic acid (six drops) were added to the mixture. The reaction was stirred in an oil bath at 60 °C for seven days. The resulting suspension was filtered under reduced pressure through a Büchner funnel and washed with a small portion of acetonitrile (30 mL). The resulting powder was dried under vacuum and characterized by spectroscopic and mass spectrometric analysis.

### 3.4. General Procedure for the Synthesis of Mn-Complexes

A 10 mL high-pressure Schlenk vessel equipped with a magnetic stirring bar and connected to the gas and vacuum line was charged with Mn(CO)_5_Br (100 mg, 3.6 × 10^−4^ mol), 1,4-diaza-1,3-butadiene (3.6 × 10^−4^ mol) and THF (6 mL). The reaction mixture was stirred overnight at room temperature. After this time, the solvent was removed under reduced pressure using a vacuum line, yielding a solid purple residue. This solid was dissolved in diethyl ether (5 mL), and the solution was decanted from the precipitate. Upon solvent evaporation, dark violet crystals were obtained. The product, isolated as a purple/navy-blue solid, was characterized by spectroscopic and mass spectrometric techniques.

### 3.5. General Procedure for Catalytic Tests

An oven-dried 5 mL glass reactor equipped with a magnetic stirring bar was charged under argon with pinacolborane (**2a**) (1.2 equiv., 30 µL, 2.07 × 10^−4^ mol), ketone (**1a**–**i**) (1 equiv., 1.72 × 10^−4^ mol), decane or dodecane (20 μL) and manganese catalyst (8.62 × 10^−6^ mol, 0.5 mol%). The reaction was carried out at 25 °C. Conversion of the substrates was monitored by GC. Reaction yields and selectivities were calculated on the basis of the GC–MS and ^1^H NMR spectra of the crude reaction mixture.

### 3.6. General Procedure for the Synthesis of Boronic Esters (**P1**–**P9**)

A 10 mL high-pressure Schlenk vessel was charged under argon with pinacolborane (**2a**) (1.2 equiv., 150 µL, 1.04 × 10^−3^ mol) and a ketone (**1a**–**i**) (1 equiv., 8.6 × 10^−4^ mol). Then, manganese catalyst **I** (0.5 mol%, 4.31 × 10^−6^ mol) was added and the reaction mixture was stirred at 25 °C. When full conversion of ketone was detected (0.5–2 h), trap-to-trap distillation was carried out. Isolated products (**P1**–**P9**) were characterized by spectroscopic methods.

### 3.7. Procedure for Hydrolysis of **P1**

A 10 mL high-pressure Schlenk vessel equipped with a magnetic stirring bar was charged with **P1** (21 mg, 1.72 × 10^−4^ mol) and diethyl ether (10 mL). Subsequently, 3 M NaOH (1 mL) and 30% H_2_O_2_ (1 mL) were added, and the reaction mixture was stirred for 1 h at 25 °C. The organic layer was extracted with diethyl ether (30 mL) and concentrated under reduced pressure. After separation of the ether layer, the aqueous layer was extracted with diethyl ether (30 mL). The combined organic phases were concentrated under reduced pressure. The crude product was purified by column chromatography on silica gel using a 1:5 mixture of ethyl acetate and *n*-hexane as eluent. Evaporation of the solvents afforded analytically pure product **P1′**.

## 4. Conclusions

To sum up, we obtained a series of bulky DABs and successfully applied them as ligands in synthesis of new manganese complexes with the generic formula [Mn(DAB)(CO)_3_Br]. The proposed Mn-species were fully characterized using various analytical techniques (NMR, UV-Vis, FT-IR, HRMS) and proven to be catalytically active in the hydroboration of ketones, leading to boronic esters. The method permits the use of no solvents, noble metal catalysts, high temperatures, long reaction times, and complicated isolation procedures, thereby establishing a synthetic pathway fully aligned with green chemistry principles. Additionally, the effectiveness of the reaction studied was demonstrated to depend on the type of DABs present in the catalysts. Complexes with bulky ligands, in contrast to those with less sterically developed ligands as well as Mn-species without DABs, require shorter times to obtain products quantitatively and ensure a slightly more efficient course of the process. The synthetic utility of the products was confirmed by performing successful hydrolysis of a selected boronic ester to its secondary alcohol, showing significant application potential [58].

## Figures and Tables

**Table 1:** Infrared spectral data of Mn complexes.

Complex	ν_(C=O)_	ν_(C=N)_	δ_(Mn-CO)_	ν_(Mn-C)_	ν_(Mn-Br)_
Mn(CO)_5_Br	2081, 2035, 1985	-	621	405	218
Mn1	2025, 1952, 1931	1626	623	405	220
I	2027, 1959, 1909	1622	621	405	224
II	2029, 1958, 1905	1619	625	407	218
III	2031, 1963, 1911	1620	621	405	220
IV	2027, 1959, 1906	1621	622	405	218
V	2025, 1948, 1917	1626	623	405	222

**Table 2:** Optimization of reaction conditions.

Entry	Catalyst	[Catalyst] (mol%)	Time (h)	Yield of P1 (%) ^[a]^
1	Mn(CO)_5_Br	0.5	3	97
2	Mn(CO)_5_Br	0.5	2	77
3	I	0.5	3	100
4	I	0.5	1	100
5	I	0.5	0.5	94
6	I	0.2	8	100
7	I	0.1	24	64
8	II	0.5	1	100
9	III	0.5	1.5	99
10	IV	0.5	1	100
11	V	0.5	1	100
12	Mn1	0.5	1	40
13	Mn1	0.5	5	61
14	Mn1	0.5	12	82
15	-	-	72	0

Reaction conditions: Solvent-free, 25 °C, [**1a**]:[**2a**] = 1:1.2, argon. ^[a]^ Determined by GC-MS analysis and confirmed by ^1^H NMR spectroscopy of the crude reaction mixture.

## Data Availability

All data generated or analyzed during this study are included in this published article (and its Supplementary Information Files: Analytical data and NMR spectra of ligands, catalysts and selected products: Figures S1–S33, XRD analysis: Tables S1 and S2, Figure S34 and S35).

## Supplementary Materials

The following supporting information can be downloaded at: https://www.mdpi.com/article/10.3390/ijms262110454/s1. Res. [59,60,61,62,63,64,65] are cited in the Supplementary Materials.
