# Supplementary material for: Manganese Complexes Bearing Bulky DAB Ligands as Efficient Catalysts for the Solvent-Free Hydroboration of Ketones
Source: Int J Mol Sci. 2025 Oct 28;26(21):10454. doi: 10.3390/ijms262110454 (PMC12607417; doi:10.3390/ijms262110454)
Supplement: Supplementary file 1 [file ijms-26-10454-s001.zip › ijms-3939383-supplementary.pdf]

Supporting Information

for

Manganese Complexes Bearing Bulky DAB Ligands as Efficient Catalysts for the Solvent-Free Hydroboration of Ketones

Aleksandra Mermela, Agata Duch, Monika Wałęsa-Chorab and Patrycja Żak*

Faculty of Chemistry, Adam Mickiewicz University in Poznań, Uniwersytetu Poznańskiego St. 8, 61-614 Poznan, Poland; aleksandra.mermela@amu.edu.pl (A.M.); agaduc@st.amu.edu.pl (A.D.); monika.walesa-chorab@amu.edu.pl (M.W.-C.)

* *Correspondence: pkw@amu.edu.pl*

CONTENTS:

1. Analytical data of isolated compounds — S-2

1.1. Analytical data of amines — S-2

1.2. Analytical data of DABs — S-3

1.3. Analytical data of Mn-complexes — S-4

1.5. Analytical data of representative products — S-5

2. NMR spectra of isolated compounds — S-6

2.1. NMR spectra of amines — S-6

2.2. NMR spectra of DABs — S-11

2.3. NMR spectra of Mn-complexes — S-16

2.3. NMR spectra of representative products — S-19

3. XRD analysis — S-23

*3.1. X-ray crystallography of the **A5**, **DAB_3** and **DAB_5** — S-23*

*3.2. X-ray crystallography of the complex **V** — S-24*

4. References — S-26

1. Analytical data of isolated compounds

1.1. Analytical data of amines

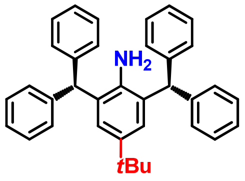 **A1**	White solid, isolated yield: 90%; ^{1}H NMR (400 MHz, $CDCl_3$, 296K): δ (ppm) 0.97 (s, 9H, $C(CH_3)_3$), 3.29 (s, 2H, NH_2), 5.46 (s, 2H, $CHPh_2$), 6.58 (s, 2H, m-C_6H_2), 7.05 – 7.12 (m, 8H, C_6H_5), 7.17 – 7.25 and 7.27 – 7.36 (m, 12H, C_6H_5); ^{13}C NMR (100 MHz, $CDCl_3$, 296K): δ (ppm) 31.30 ($C(CH_3)_3$), 33.94 ($C(CH_3)_3$), 52.70 ($CHPh_2$), 125.49, 126.55, 128.39, 128.53, 129.52, 139.50, 140.02, 142.88; MS (ESI+): m/z: 482 $[M+H]^+$.
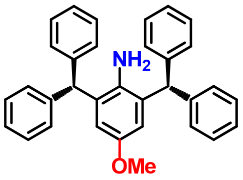 **A2**	White solid, isolated yield: 88%; ^{1}H NMR (400 MHz, $CDCl_3$, 296K): δ (ppm): 3.16 (s, 2H, NH_2), 3.45 (s, 3H, OCH_3), 5.50 (s, 2H, $CHPh_2$), 6.22 (s, 2H, m-CH_{Ar}), 7.09 (d, 8H, J_{HH} = 7.6 Hz, Ph), 7.24 (d, 4H, J_{HH} = 7.6 Hz, Ph), 7.28-7.32 (m, 8H, Ph); ^{13}C NMR (100 MHz, $CDCl_3$, 296K): δ (ppm): 52.50 ($CHPh_2$), 55.13 (OCH_3), 114.38, 126.70, 128.52, 129.50, 130.82, 135.89, 142.49, 151.82; MS (ESI+): m/z 456 $[M+H]^+$.
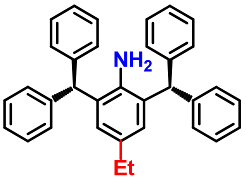 **A3**	White solid, isolated yield: 84%; ^{1}H NMR (400 MHz, $CDCl_3$, 296K): δ (ppm) 0.95 (t, 3H, J_{HH} = 7.6 Hz, CH_3CH_2), 2.32 (q, 2H, J_{HH} = 7.6 Hz, CH_3CH_2), 3.29 (s, 2H, NH_2), 5.47 (s, 2H, $CHPh_2$), 6.42 (s, 2H, m-CH_{Ar}), 7.07 – 7.14 (m, 8H, Ph), 7.20 – 7.25 (m, 4H, Ph), 7.27 – 7.32 (m, 8H, Ph); ^{13}C NMR (100 MHz, $CDCl_3$, 296K): δ (ppm): 15.73 (CH_3CH_2), 28.16 (CH_3CH_2), 52.44 ($CHPh_2$), 55.97 ($CHPh_2$), 126.52, 127.79, 128.40, 129.11, 129.50, 133.18, 139.79, 142.76; MS (ESI+): m/z 454 $[M+H]^+$.
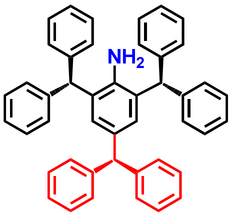 **A4**	White solid, isolated yield: 81%; ^{1}H NMR (400 MHz, $CDCl_3$, 296K): δ (ppm): 3.37 (s, 2H, NH_2), 5.18 (s, 1H, $CHPh_2$), 5.43 (s, 2H, $CHPh_2$), 6.38 (s, 2H, m-CH_{Ar}), 6.83 – 7.26 (m, 30H, Ph); ^{13}C NMR (100 MHz, $CDCl_3$, 296K): δ (ppm): 52.41 ($CHPh_2$), 55.97 ($CHPh_2$), 125.64, 126.48, 127.87, 128.37, 128.85, 129.04, 129.34, 129.71, 132.38, 140.29, 142.51, 144.75; MS (ESI+): m/z 592 $[M+H]^+$.
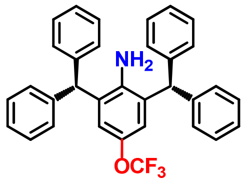 **A5**	White solid, isolated yield: 79%; ^{1}H NMR (400 MHz, $CDCl_3$, 296K): δ (ppm): 3.74 (s, 2H, NH_2), 5.40 (s, 2H, $CHPh_2$), 6.82 (s, 2H, m-CH_{Ar}), 7.07 (d, 8H, J_{HH} = 7.0 Hz, Ph), 7.20-7.25 (m, 4H, Ph), 7.27-7.34 (m, 8H, Ph); ^{13}C NMR (100 MHz, $CDCl_3$, 296K): δ (ppm): 52.34 ($CHPh_2$), 121.56 (q, J = 32.1 MHz, OCF_3), 125.47, 127.02, 128.72, 129.36, 130.26, 140.88, 141.71, 145.21; MS (ESI+): m/z 456 $[M+H]^+$.

1.2. Analytical data of DABs

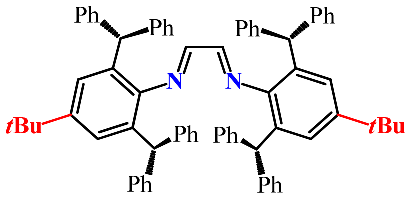 **DAB_1**	Yellow solid, isolated yield: 80%; ^{1}H NMR (400 MHz, $CDCl_3$, 296K): δ (ppm): 0.97 (s, 18H, C(CH_3)$_3$), 5.46 (s, 4H, C*H*Ph_2), 6.89 (s, 4H, m-CH_{Ar}), 6.98-7.00 (m, 12H, Ph + NC*H*), 7.13-7.25 (m, 30H, Ph); ^{13}C NMR (100 MHz, $CDCl_3$, 296K): δ (ppm): 31.13 (*C*(CH_3)$_3$), 34.33 (C(CH_3)$_3$), 51.31 (*C*HPh_2), 125.50, 126.21, 128.15, 129.47, 131.33, 143.99, 146.55, 163.76 (N*C*H); MS (ESI+): m/z 1007 $[M+Na]^+$.
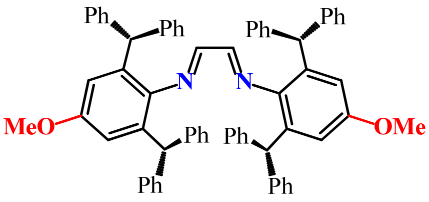 **DAB_2**	Yellow solid, isolated yield: 90%; ^{1}H NMR (400 MHz, $CDCl_3$, 296K): δ (ppm): 3.54 (s, 6H, OCH_3), 5.29 (s, 4H, C*H*Ph_2), 6.45 (s, 4H, m-CH_{Ar}), 7.02 (d, 16H, J_{HH} = 6.8 Hz, Ph), 7.13-7.23 (m, 26H, Ph + NC*H*); ^{13}C NMR (100 MHz, $CDCl_3$, 296K): δ (ppm): 51.22 (*C*HPh_2), 55.05 (OCH_3), 114.16, 126.42, 128.30, 129.46, 133.71, 143.56, 156.16, 164.17 (N*C*H); MS (ESI+): m/z 956 $[M+Na]^+$.
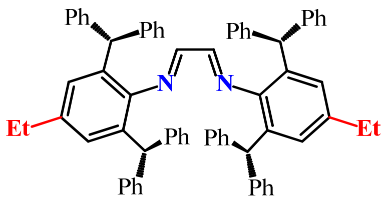 **DAB_3**	Yellow solid, isolated yield: 86%; ^{1}H NMR (400 MHz, $CDCl_3$, 296K): δ (ppm): 1.03 (t, 6H, J_{HH} = 7.6 Hz, CH_3CH_2), 2.42 (q, 4H, J_{HH} = 7.6 Hz, CH_3CH_2), 5.26 (s, 4H, C*H*Ph_2), 6.70 (s, 4H, m-CH_{Ar}), 6.99 – 7.01 (m, 12H, Ph + NC*H*), 7.16 – 7.25 (m, 30H, Ph); ^{13}C NMR (100 MHz, $CDCl_3$, 296K): δ (ppm): 16.73 (CH_3CH_2), 28.31 (CH_3CH_2), 53.44 (*C*HPh_2), 56.97 (*C*HPh_2), 126.52, 127.79, 129.11, 129.50, 133.18, 139.79, 142.76, 163.76 (N*C*H); MS (ESI+): m/z 951 $[M+Na]^+$.
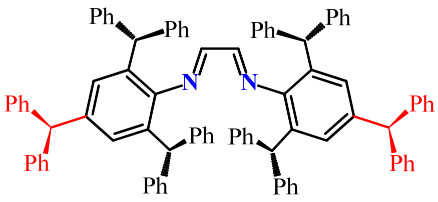 **DAB_4**	Yellow solid, isolated yield: 82%; ^{1}H NMR (400 MHz, $CDCl_3$, 296K): δ (ppm): 5.23 (s, 4H, C*H*Ph_2), 5.30 (s, 2H, C*H*Ph_2), 6.66 (s, 4H, m-CH_{Ar}), 6.88-6.93 (m, 22H, Ph + NC*H*), 7.10-7.24 (m, 40H, Ph); ^{13}C NMR (100 MHz, $CDCl_3$, 296K): δ (ppm): 51.07 (*C*HPh_2), 56.04 (*C*HPh_2), 125.98, 126.18, 128.02, 128.14, 129.08, 129.31, 129.76, 131.91, 139.34, 143.61, 143.94, 147.19, 163.79 (N*C*H); MS (ESI+): m/z 1228 $[M+Na]^+$.
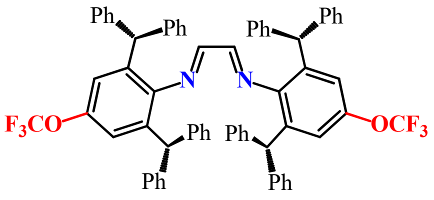 **DAB_5**	Yellow solid, isolated yield: 71%; ^{1}H NMR (400 MHz, $CDCl_3$, 296K): δ (ppm): 5.19 (s, 4H, C*H*Ph_2), 6.72 (s, 4H, m-CH_{Ar}), 6.93-6.97 (m, 16H, Ph), 7.07 (s, 2H, NC*H*) 7.17-7.24 (m, 24H, Ph); ^{13}C NMR (100 MHz, $CDCl_3$, 296K): δ (ppm): 51.16 (*C*HPh_2), 121.27 (q, OCF_3), 126.81, 128.52, 129.32, 134.13, 142.79, 145.78, 147.20, 163.96 (N*C*H); MS (ESI+): m/z 1063 $[M+Na]^+$.

1.3. Analytical data of MN-complexes

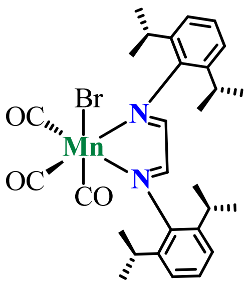 **Mn1**	Dark violets crystalline solid, isolated yield: 87%; ^{1}H NMR (600 MHz, $CDCl_3$, 296K): δ (ppm): 1.17 (s, 12H, CH(C*H*$_3$)$_2$), 1.92 (s, 12H, CH(C*H*$_3$)$_2$), 2.69 (s, 2H, C*H*Ph), 3.85 (s, 2H, C*H*Ph), 7.22-7.40 (m, 8H, Ph), 8.16 (s, 2H, NC*H*); UV-VIS (DCM, nm): *λmax* 365, 582; FT-IR (cm^{-1}): $\nu_{(C=O)}$ 2025, 1952, 1931, $\nu_{(C=N)}$ 1626, $\delta_{(Mn\text{-}CO)}$ 623, $\nu_{(Mn\text{-}C)}$ 405, $\nu_{(Mn\text{-}Br)}$ 220; MS (FD) m/z 515 $[M-Br]^+$; HRMS (FD): calcd. for $C_{29}H_{36}MnN_2O_3$: 515.210; found: 515.210; E.A. calcd. for $C_{29}H_{36}BrMnN_2O_3$: C: 58.50, H: 6.09, N: 4.70; found: C: 58.52, H: 6.14, N: 4.71. The analytical data are in agreement with the literature. [S1]
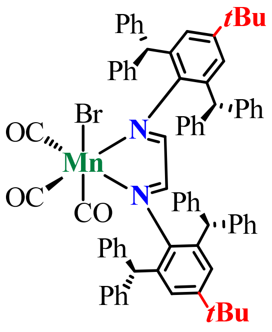 **I**	Dark violets crystalline solid, isolated yield: 80%; ^{1}H NMR (600 MHz, $CDCl_3$, 296K): δ (ppm): 1.07 (s, 18H, C(C*H*$_3$)$_3$), 5.28 (s, 2H, C*H*Ph$_2$), 5.70-5.92 (m, 2H, C*H*Ph$_2$), 6.27 (s, 2H, m-C*H*$_{Ar}$), 6.69 (s, 2H, m-C*H*$_{Ar}$), 6.75-7.08 (m, 22H, Ph + NC*H*), 7.11-7.23 (m, 15H, Ph), 7.31-7.36 (m, 5H, Ph); UV-VIS (DCM, nm): *λmax* 361, 581; FT-IR (cm^{-1}): $\nu_{(C=O)}$ 2027, 1959, 1909, $\nu_{(C=N)}$ 1622, $\delta_{(Mn\text{-}CO)}$ 621, $\nu_{(Mn\text{-}C)}$ 405, $\nu_{(Mn\text{-}Br)}$ 224; MS (FD) m/z 1123 $[M-Br]^+$; HRMS (FD): calcd. for $C_{77}H_{68}MnN_2O_3$: 1123.4610; found: 1123.4570; E.A. calcd. for $C_{77}H_{68}BrMnN_2O_3$: C: 76.80, H: 5.69, N: 2.33; found: C: 76.85, H: 5.70, N: 2.30.
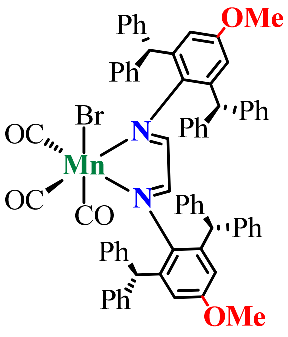 **II**	Dark violets crystalline solid, isolated yield: 90%; ^{1}H NMR (600 MHz, $CDCl_3$, 296K): δ (ppm): 3.50 (s, 6H, OC*H*$_3$), 5.25 (s, 2H, C*H*Ph), 5.63 (s, 1H, C*H*Ph), 5.82 (s, 1H, C*H*Ph), 6.27 (s, 2H, m-C*H*$_{Ar}$), 6.37 – 6.52 (m, 4H, m-C*H*$_{Ar}$) 6.69 (s, 2H, NC*H*), 6.81 – 7.34 (m, 40H, Ph); UV-VIS (DCM, nm): *λmax* 380, 563; FT-IR (cm^{-1}): $\nu_{(C=O)}$ 2029, 1958, 1905, $\nu_{(C=N)}$ 1619, $\delta_{(Mn\text{-}CO)}$ 625, $\nu_{(Mn\text{-}C)}$ 407, $\nu_{(Mn\text{-}Br)}$ 218; MS (FD) m/z 1071 $[M-Br]^+$; HRMS (FD): calcd. for $C_{71}H_{56}MnN_2O_5$: 1071.3570; found: 1071.3573; E.A. calcd. for $C_{71}H_{56}BrMnN_2O_5$: C: 74.02, H: 4.90, N: 2.43; found: C: 74.09, H: 4.96, N: 2.42.
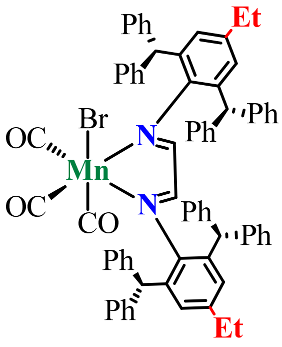 **III**	Dark violets crystalline solid, isolated yield: 84%; ^{1}H NMR (600 MHz, CD_2Cl_2, 296K): δ (ppm): 0.92-1.04 (m, 6H, C*H*$_3$CH$_2$), 2.33-2.53 (m, 4H, CH$_3$C*H*$_2$), 5.23 (s, 2H, C*H*Ph$_2$), 5.69-5.84 (m, 1H, C*H*Ph$_2$), 6.18-6.28 (m, 1H, C*H*Ph$_2$), 6.42 (s, 1H, m-C*H*$_{Ar}$), 6.64-6.68 (m, 1H, m-C*H*$_{Ar}$), 6.71 (m, 2H, m-C*H*$_{Ar}$), 6.79 – 6.85 (m, 2H, NC*H*), 6.97-6.99 (m, 8H, Ph), 7.08-7.11 (m, 7H, Ph), 7.16-7.25 (m, 20H, Ph), 7.28-7.33 (m, 5H, Ph); UV-VIS (DCM, nm): *λmax* 365, 578; FT-IR (cm^{-1}): $\nu_{(C=O)}$ 2031, 1963, 1911, $\nu_{(C=N)}$ 1620, $\delta_{(Mn\text{-}CO)}$ 621, $\nu_{(Mn\text{-}C)}$ 405, $\nu_{(Mn\text{-}Br)}$ 218; MS (FD) m/z 1067 $[M-Br]^+$; E.A. calcd. for $C_{73}H_{60}BrMnN_2O_3$: C: 76.37, H: 5.27, N: 2.44; found: C: 76.39, H: 5.29, N: 2.45.

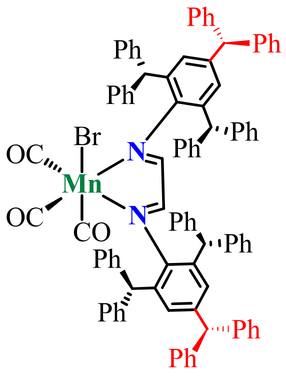<br>**IV**	Dark violets crystalline solid, isolated yield: 82%; ^{1}H NMR (600 MHz, $CDCl_3$, 296K): δ (ppm): 5.20 (s, 2H, C*H*Ph_2), 5.60–5.96 (m, 2H, C*H*Ph_2), 6.21 (s, 2H, C*H*Ph_2), 6.36 (s, 1H, m-CH_{Ar}), 6.56-6.67 (m, 3H, m-CH_{Ar}), 6.69-7.56 (m, 62H, Ph + NC*H*); UV-VIS (DCM, nm): *λmax* 365, 578; FT-IR (cm^{-1}): $\nu_{(C=O)}$ 2027, 1959, 1906, $\nu_{(C=N)}$ 1621, $\delta_{(Mn\text{-}CO)}$ 622, $\nu_{(Mn\text{-}C)}$ 405, $\nu_{(Mn\text{-}Br)}$ 218; MS (FD) m/z 1503 $[M+Br]^-$; E.A. calcd. for $C_{95}H_{72}BrF_6MnN_2O_3$: C: 80.10, H: 5.09, N: 1.97; found: C: 80.16, H: 5.12, N: 1.98.
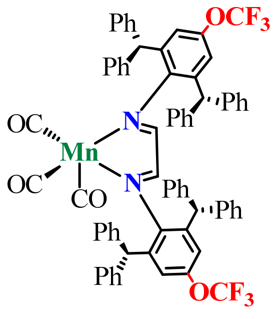<br>**V**	Dark violets crystalline solid, isolated yield: 77%; ^{1}H NMR (600 MHz, CD_2Cl_2, 296K): δ (ppm): 5.19 (s, 2H, C*H*Ph_2), 5.72 (s, 1H, C*H*Ph_2), 5.83 (s, 1H, C*H*Ph_2), 6.22 (s, 2H, NC*H*), 6.55-6.72 (m, 4H, m-CH_{Ar}), 6.91-7.34 (m, 40H, Ph); UV-VIS (DCM, nm): *λmax* 374, 560; FT-IR (cm^{-1}): $\nu_{(C=O)}$ 2025, 1948, 1917, $\nu_{(C=N)}$ 1626, δ($_{Mn\text{-}CO)}$ 623, $\nu_{(Mn\text{-}C)}$ 405, $\nu_{(Mn\text{-}Br)}$ 222; MS (FD) m/z 1179 $[M\text{-}Br]^+$; E.A. calcd. for $C_{71}H_{50}BrF_6MnN_2O_5$: C: 67.68, H: 4.00, N: 2.22; found: C: 67.70, H: 4.05, N: 2.25.

1.4. Analytical data of representative products

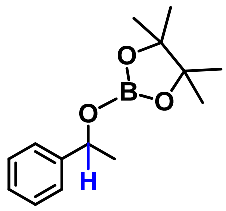<br>**P1**	Colorless liquid, isolated yield: 98%; ^{1}H NMR (400 MHz, $CDCl_3$, 296K): δ (ppm): 1.20-1.27 (m, 12H, CH_3), 1.51 (d, 3H, J_{HH} = 6.5 Hz, CH_3), 5.26 (q, 1H, J_{HH} = 6.5 Hz, OC*H*), 7.23 – 7.25 (m, 1H, C_6H_5), 7.30 – 7.35 (m, 2H, C_6H_5), 7.36-7.39 (m, 2H, C_6H_5); ^{13}C NMR (100 MHz, $CDCl_3$, 296K): δ (ppm): 24.43 (4CH_3), 25.35 (CH_3), 72.47 (OCH), 82.63 (-B-OCHpin), 125.22, 126.99, 128.08, 144.46. These data matched those reported in the literature.[S2]
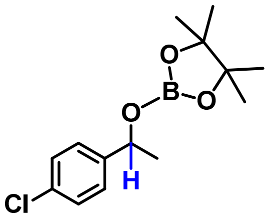<br>**P1**	Colorless liquid, isolated yield: 97%; ^{1}H NMR (400 MHz, $CDCl_3$, 296K): δ (ppm): 1.81-1.23 (m, 12H, CH_3), 1.44 (d, 3H, J_{HH} = 6.5 Hz, CH_3), 5.19 (q, 1H, J_{HH} = 6.5 Hz, OC*H*), 7.24 – 7.25 (m, 1H, C_6H_5), 7.27-7.30 (m, 3H, C_6H_5); ^{13}C NMR (100 MHz, $CDCl_3$, 296K): δ (ppm): 24.43 (4CH_3), 25.24 (CH_3), 71.86 (OCH), 82.79 (-B-OCHpin), 126.68, 128.22, 129.65, 142.99. These data matched those reported in the literature.[S3]

**P8**	Colorless liquid, isolated yield: 98%; ^{1}H NMR (400 MHz, CDCl$_3$, 296K): δ (ppm): 0.86 (t, 3H, J_{HH} = 7.1 Hz, CH_3), 1.15 (d, 3H, J_{HH} = 6.2 Hz, CH_3), 1.21-1.29 (m, 18H, CH_3 and CH_2), 1.32-1.40 (m, 2H, CH_2), 4.13 (dd, 1H, J_{HH} = 12.3, 6.2 Hz, OCH); ^{13}C NMR (100 MHz, CDCl$_3$, 296K): δ (ppm): 13.97 (CH$_3$), 22.51 (CH$_2$), 22.58 (CH$_2$), 24.47 (4CH$_3$), 25.13 (CH$_3$), 31.68 (CH$_2$), 38.09 (CH$_2$), 70.84 (OCH), 82.32 82.63 (-B-OCHpin). These data matched those reported in the literature.[S3]
**P1'**	Colorless liquid, isolated yield: 90%; ^{1}H NMR (400 MHz, CDCl$_3$, 296K): δ (ppm): 1.51 (d, 3H, J_{HH} = 6.7 Hz, CH_3), 3.95 (br s, 1H, OH), 4.85 (q, 1H, J_{HH} = 6.7 Hz, (HC(CH$_3$)), 7.32-7.35 (m, 1H, C$_6$$H_5$), 7.37-7.44 (m, 4H, C$_6$$H_5$); ^{13}C NMR (100 MHz, CDCl$_3$, 296K): δ (ppm): 24.78 (CH$_3$), 69.62 (CH), 152.14, 126.84, 127.98, 145.63; MS: *m/z* (rel. intensity): 43 (21), 51 (26), 63 (7), 79 (87), 91 (2), 107 (100), 122 (12, M$^+$). The analytical data are in agreement with the literature.[S3]

2. NMR spectra of compounds

2.1. NMR spectra of amines

A1

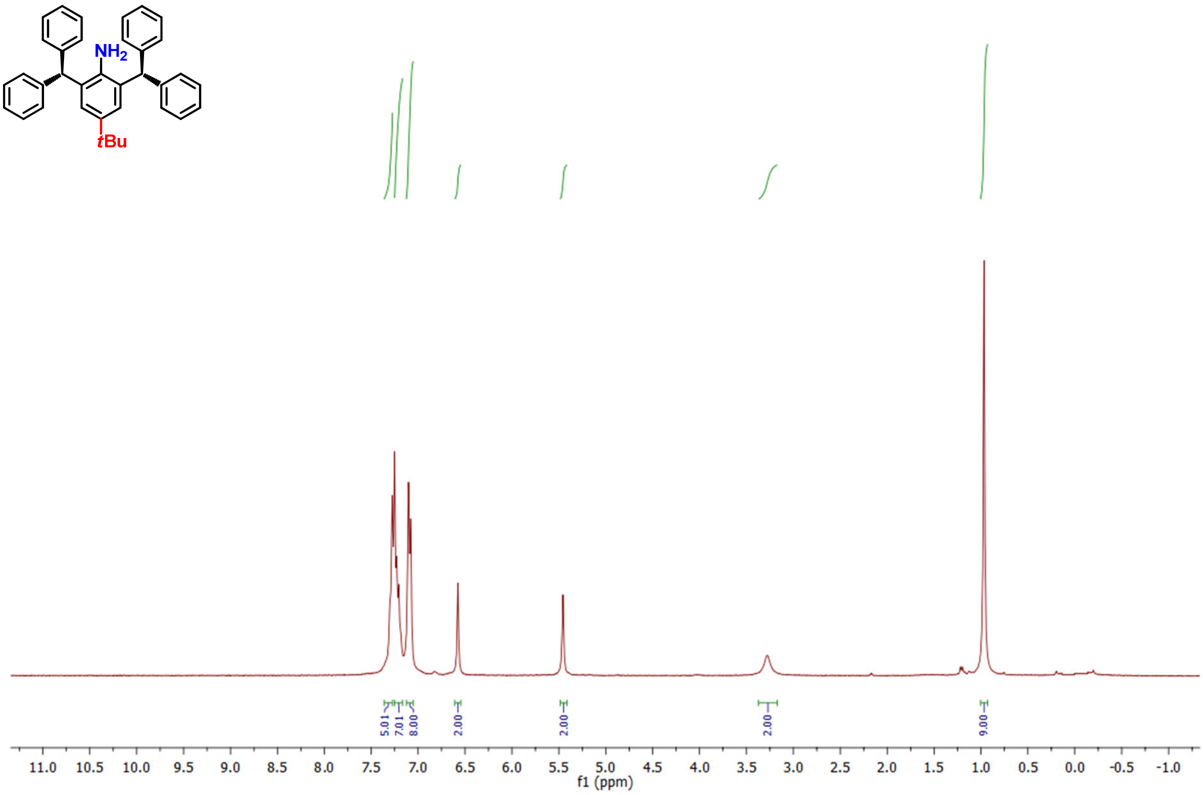

Figure S1. ^{1}H NMR (400 MHz, CDCl$_3$) of amine **A1**

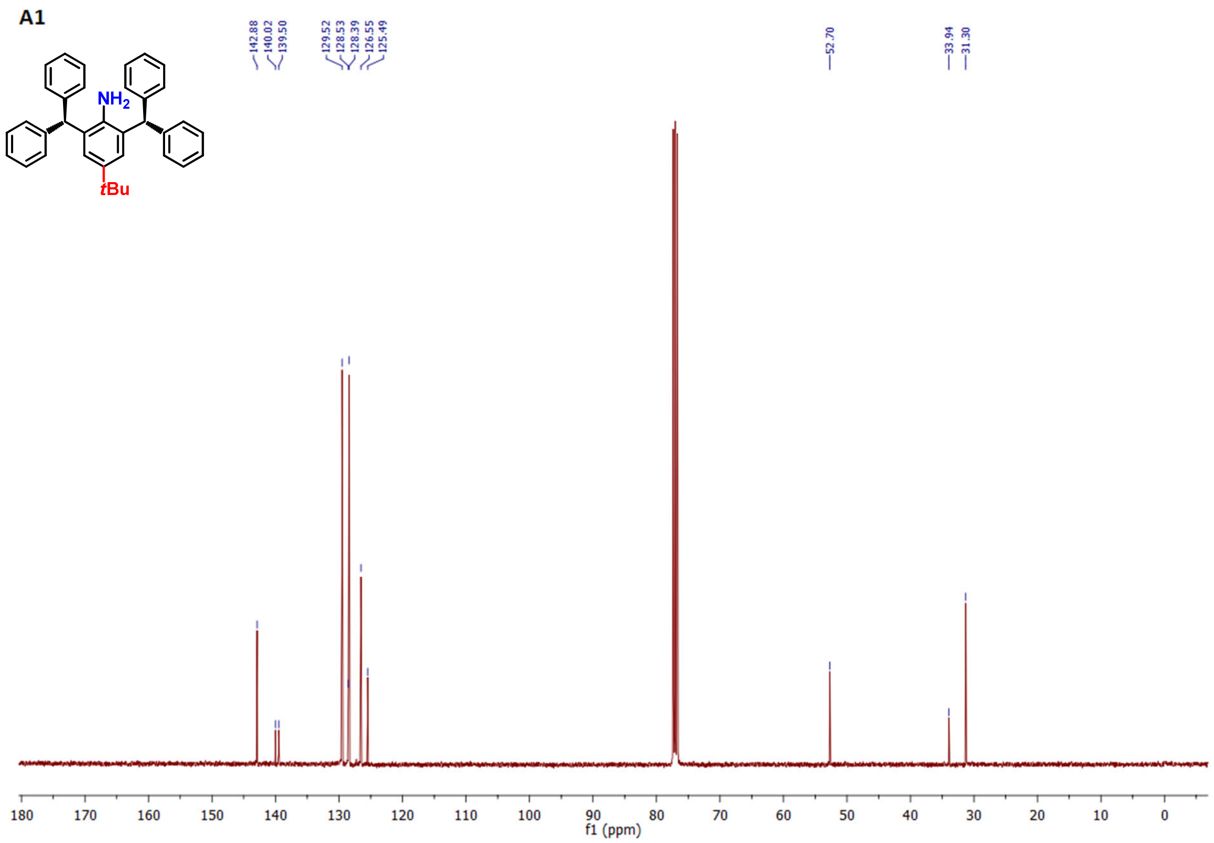

Figure S2. ^{13}C NMR (101 MHz, $CDCl_3$) of amine **A1**

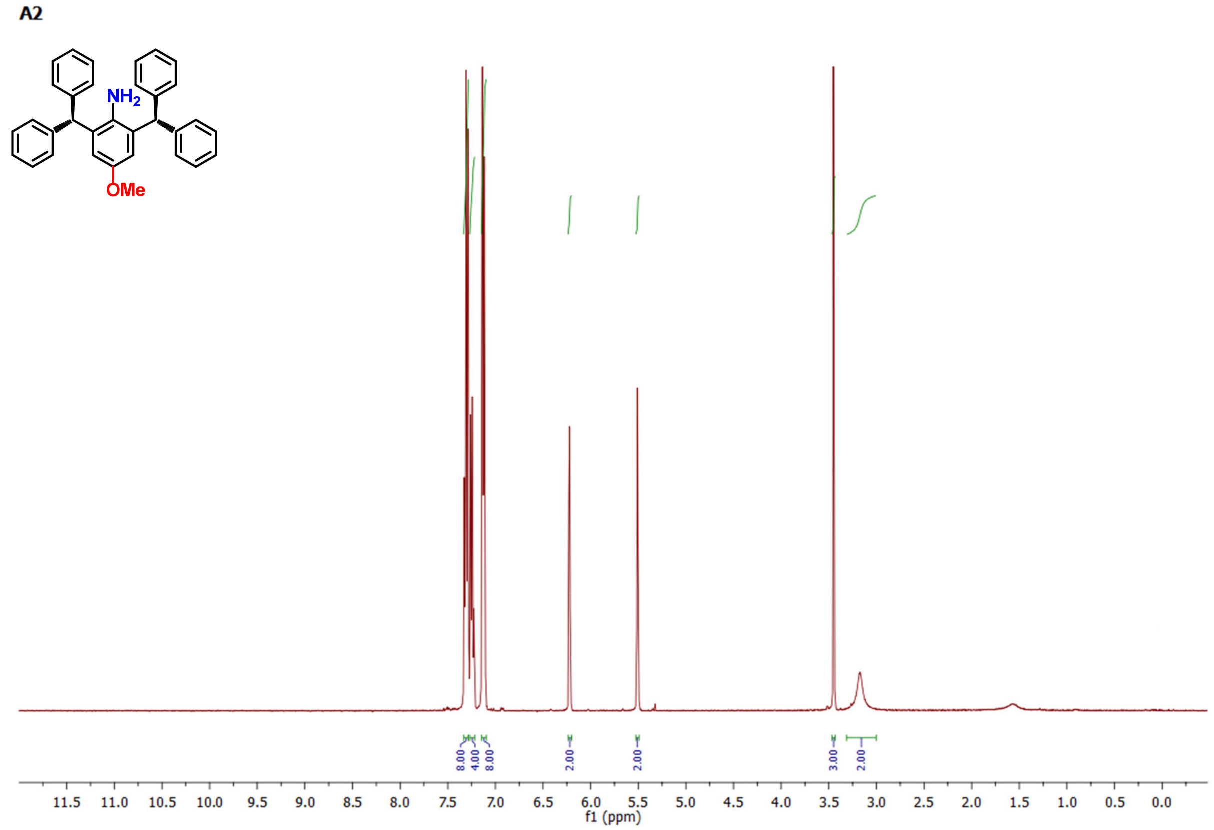

Figure S3. ^{1}H NMR (400 MHz, $CDCl_3$) of amine **A2**

A2

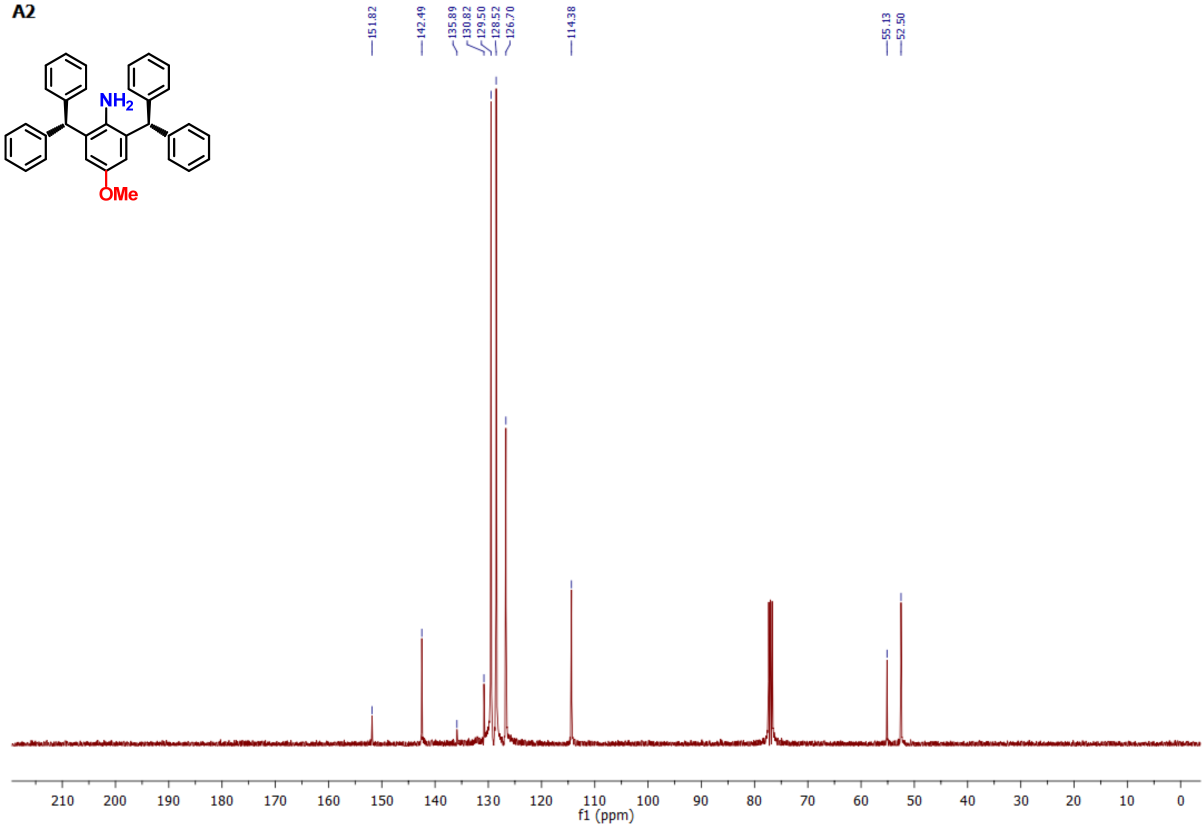

Figure S4. ^{13}C NMR (101 MHz, $CDCl_3$) of amine **A2**

A3

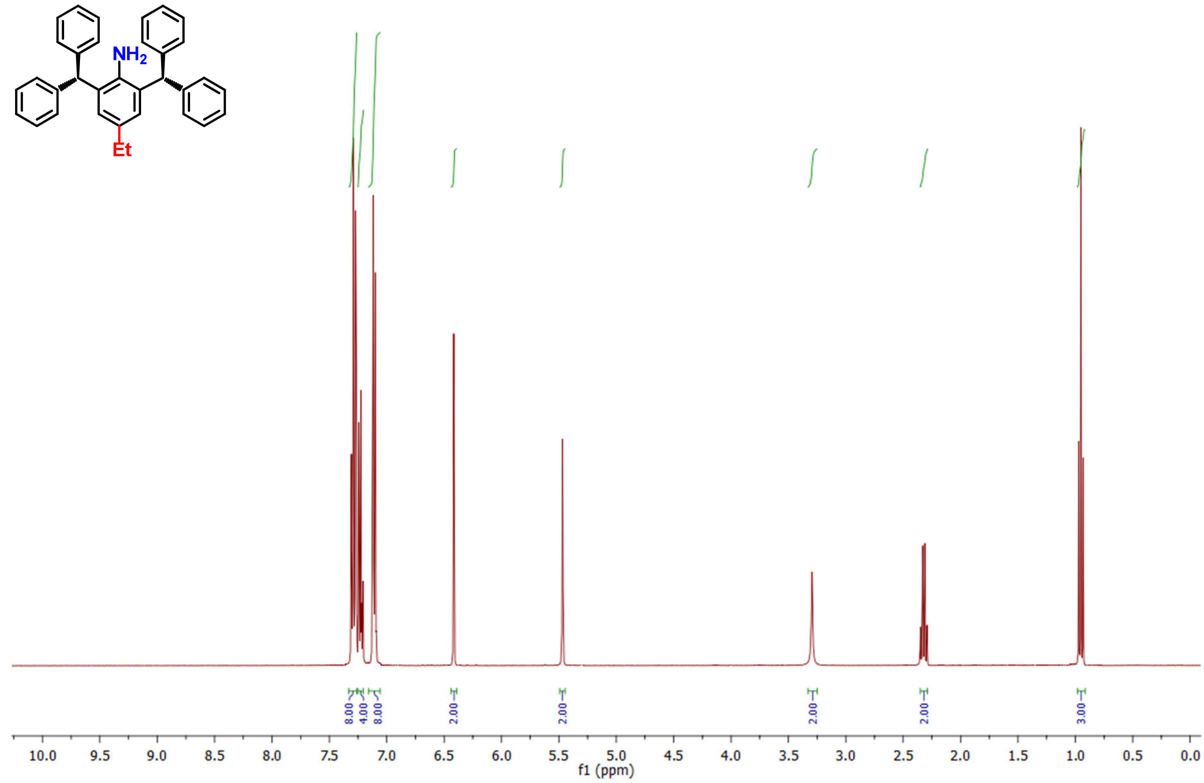

Figure S5. ^{1}H NMR (400 MHz, $CDCl_3$) of amine **A3**

A3

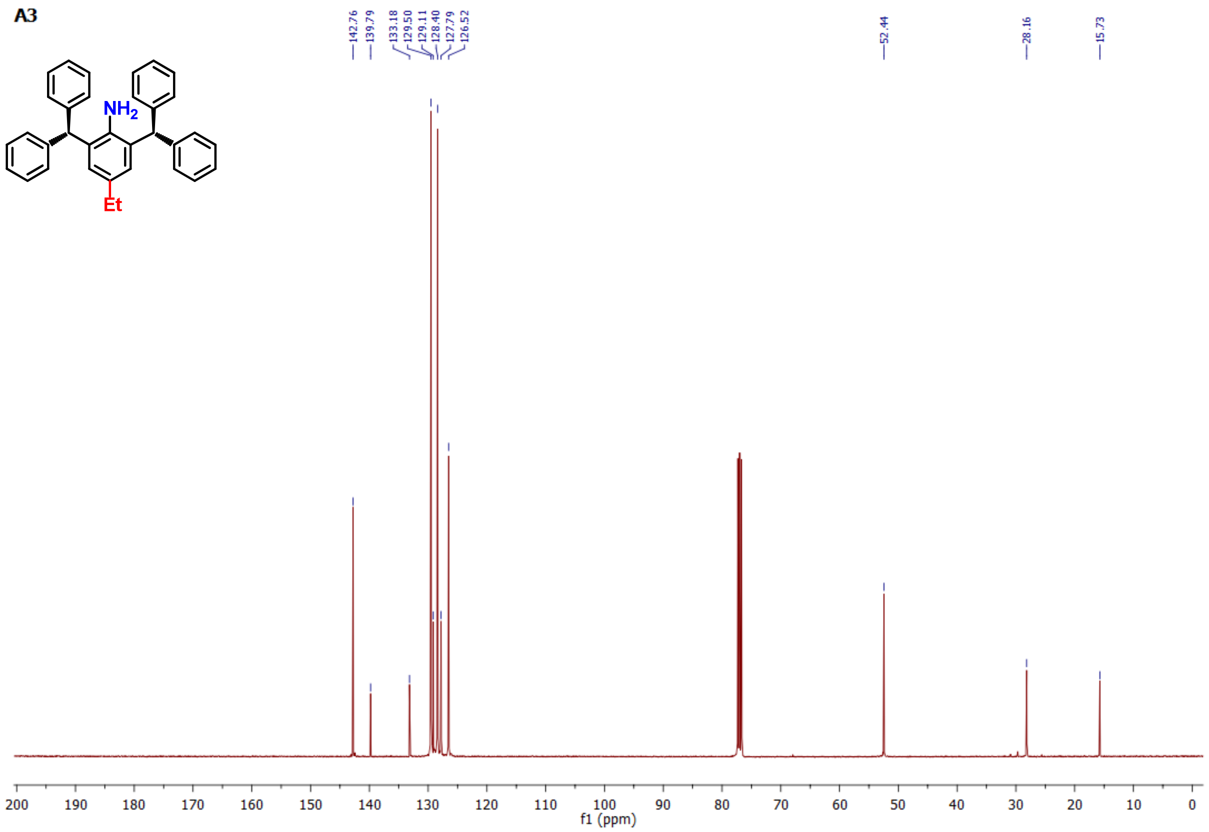

Figure S6. ^{13}C NMR (101 MHz, $CDCl_3$) of amine **A3**

A4

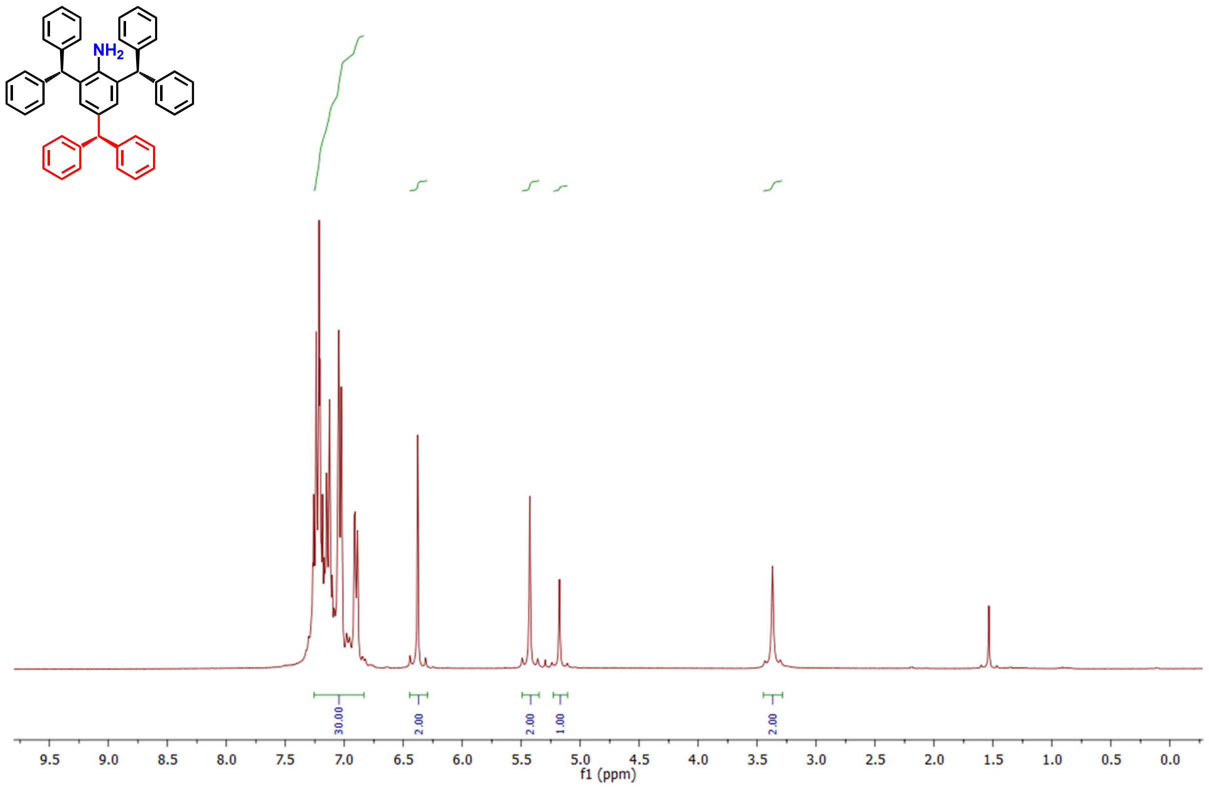

Figure S7. ^{1}H NMR (400 MHz, $CDCl_3$) of amine **A4**

A4

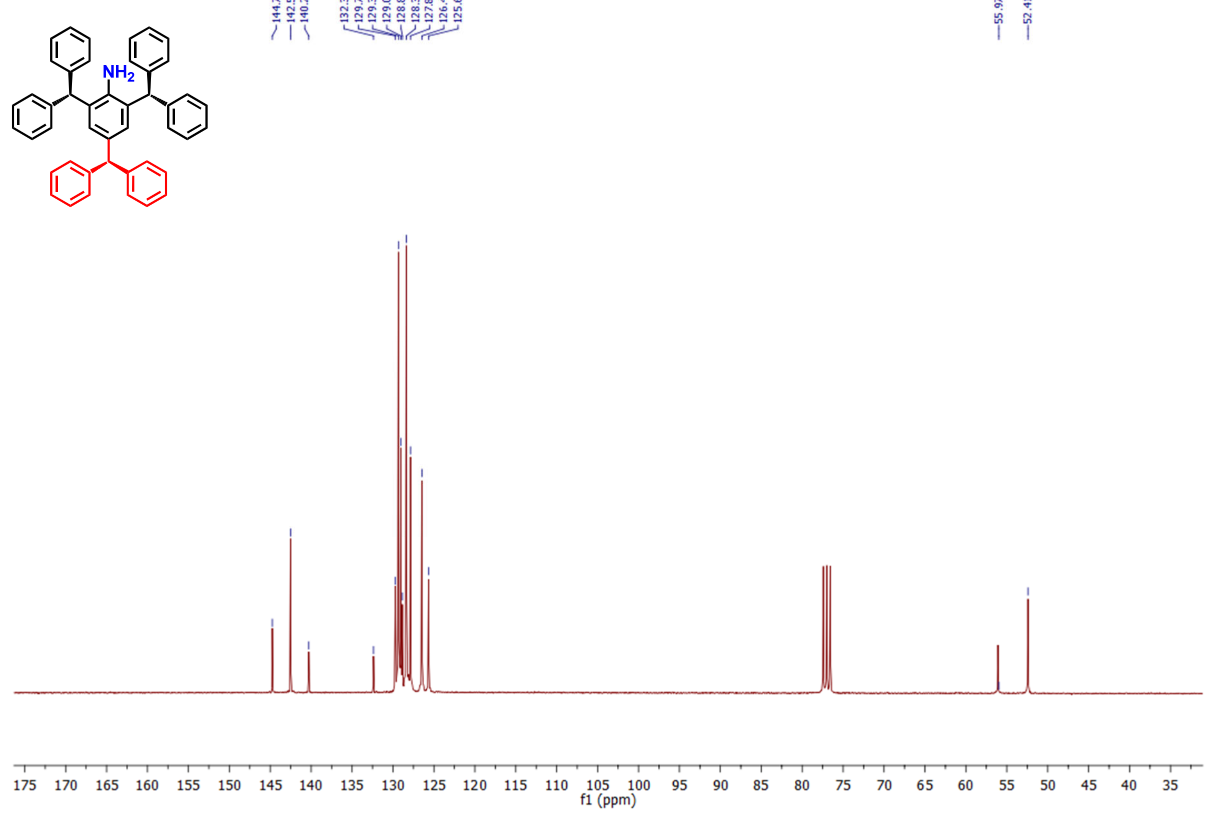

Figure S8. ^{13}C NMR (101 MHz, $CDCl_3$) of amine **A4**

A5

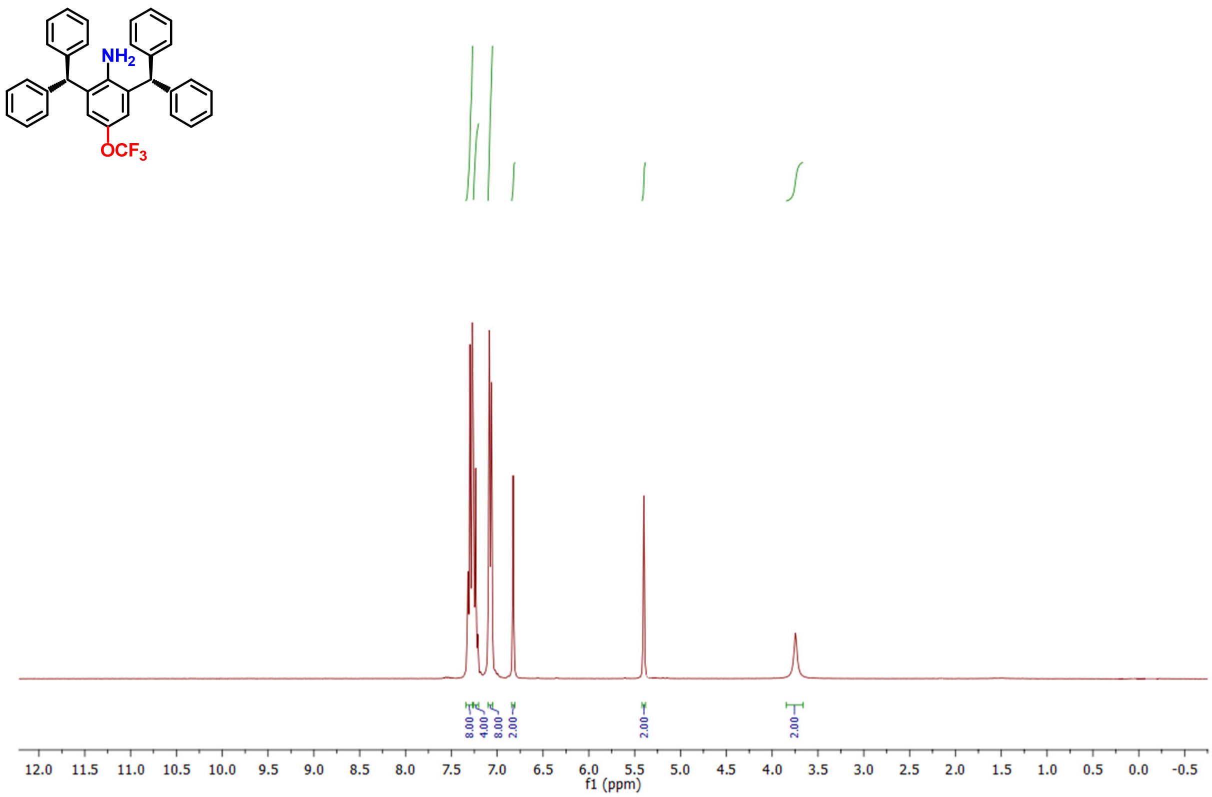

Figure S9. ^{1}H NMR (400 MHz, $CDCl_3$) of amine **A5**

A5

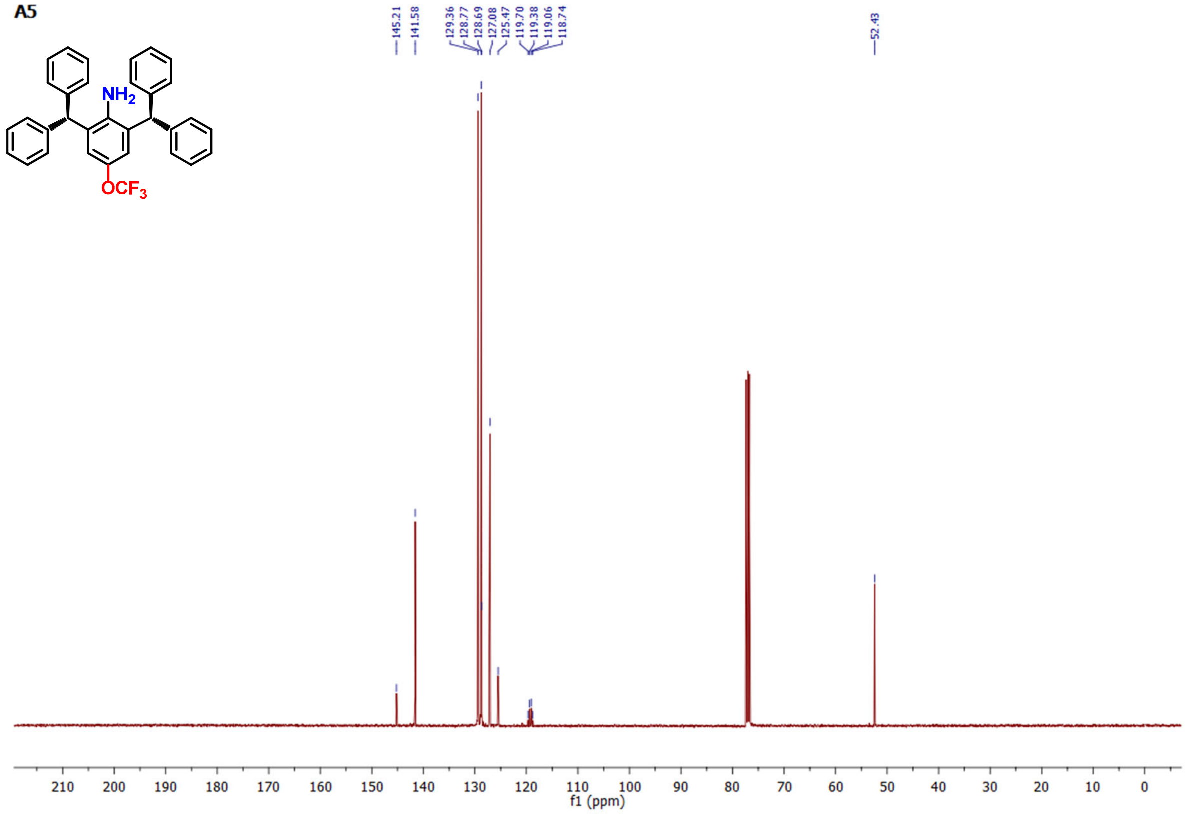

Figure S10. ^{13}C NMR (101 MHz, $CDCl_3$) of amine **A5**

2.2. NMR spectra of DABs

DAB_1

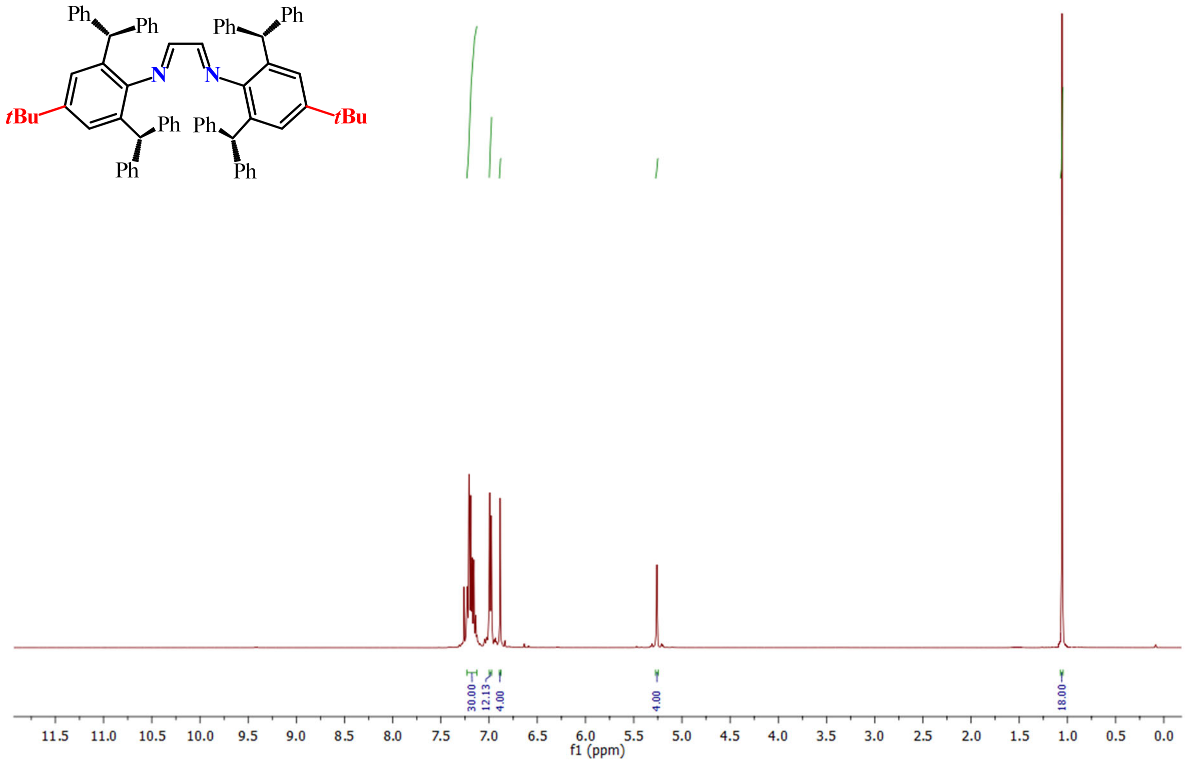

Figure S11. ^{1}H NMR (400 MHz, $CDCl_3$) of **DAB_1**

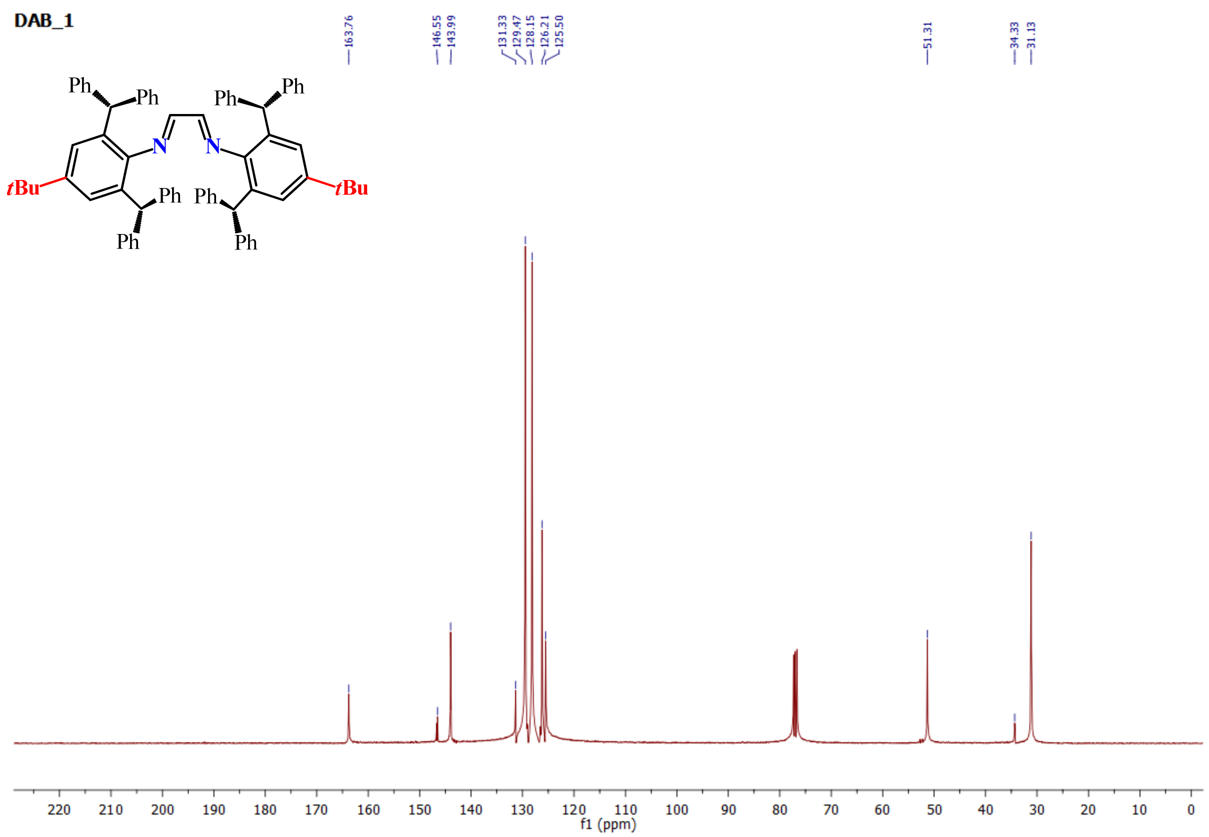

Figure S12. ^{13}C NMR (101 MHz, $CDCl_3$) of **DAB_1**

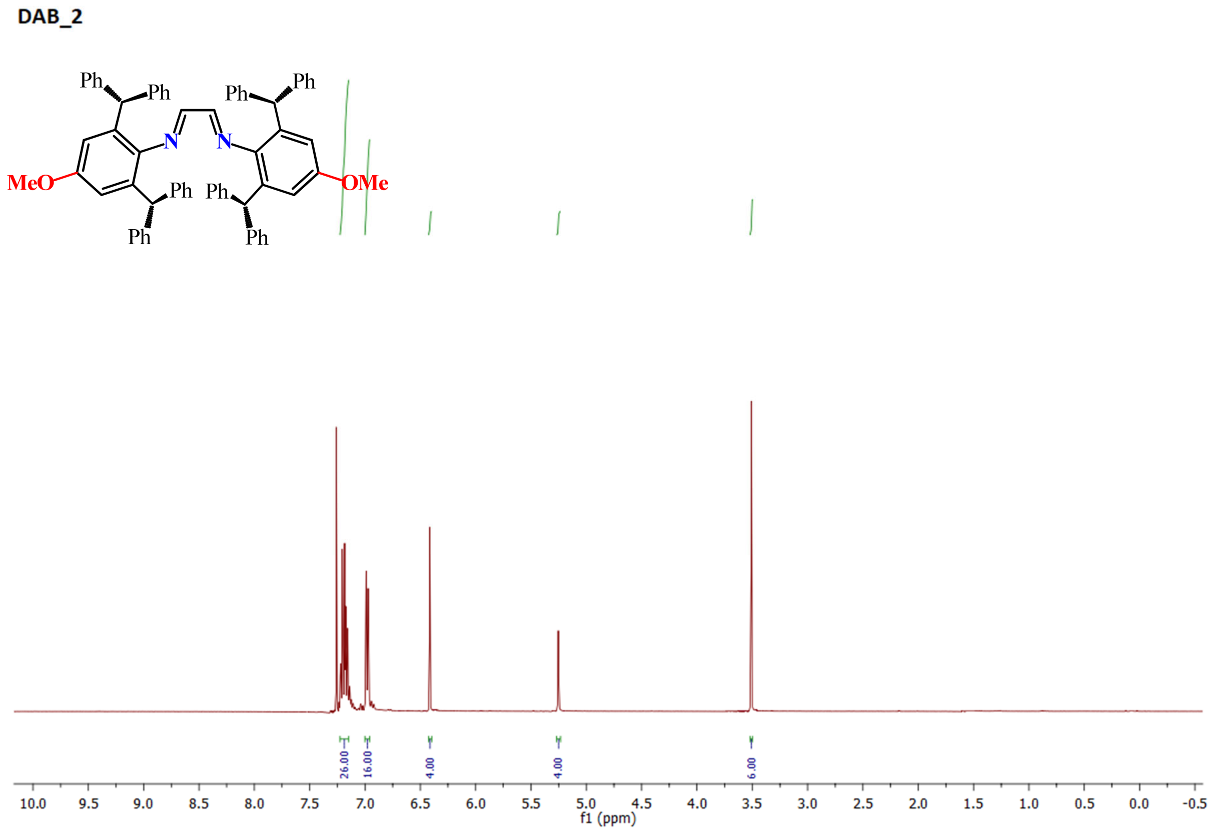

Figure S13. ^{1}H NMR (400 MHz, $CDCl_3$) of **DAB_2**

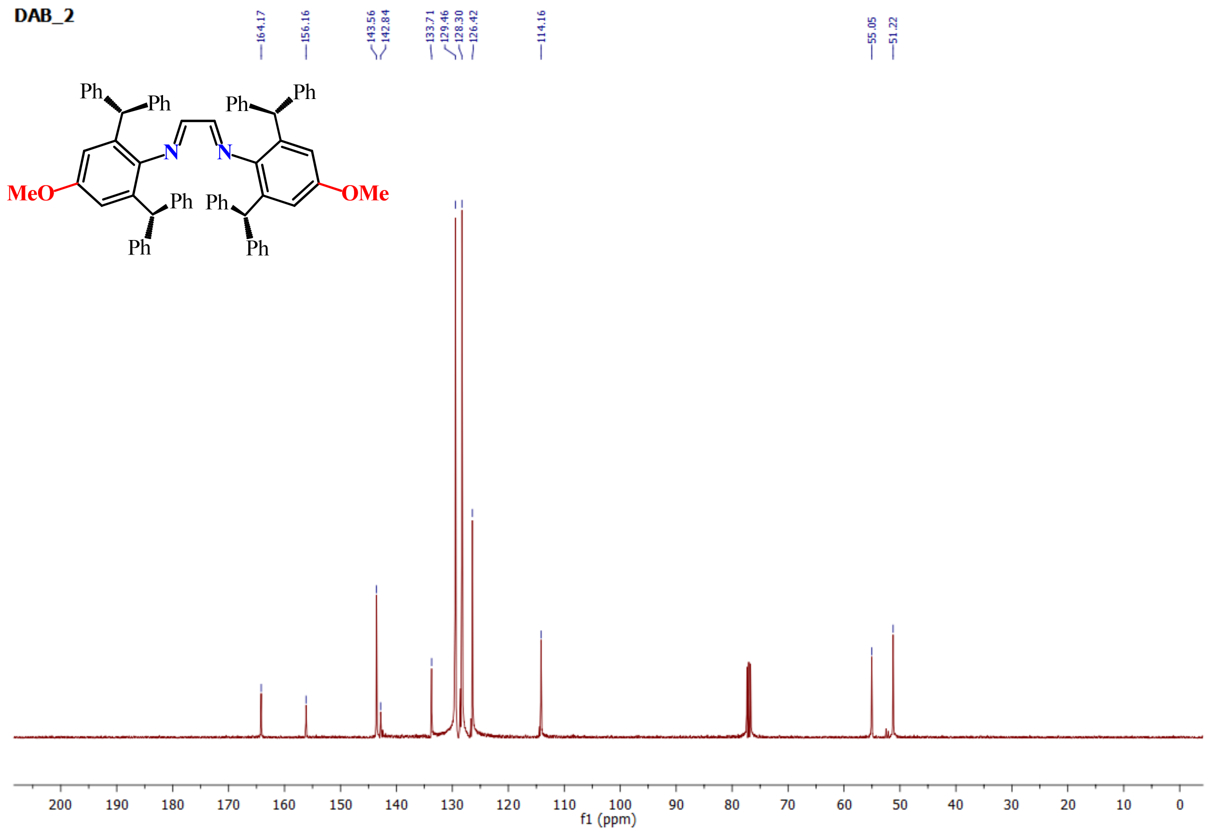

Figure S14. ^{13}C NMR (101 MHz, $CDCl_3$) of **DAB_2**

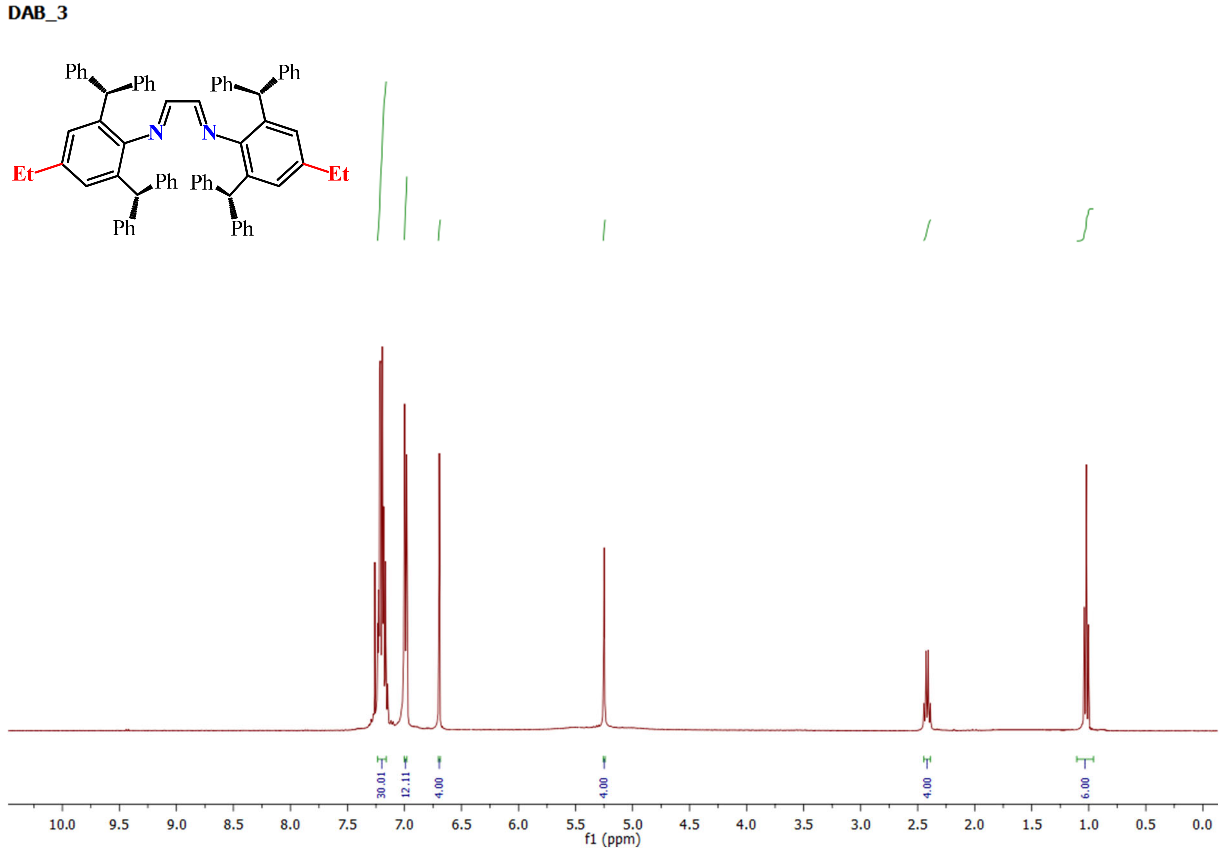

Figure S15. ^{1}H NMR (400 MHz, $CDCl_3$) of **DAB_3**

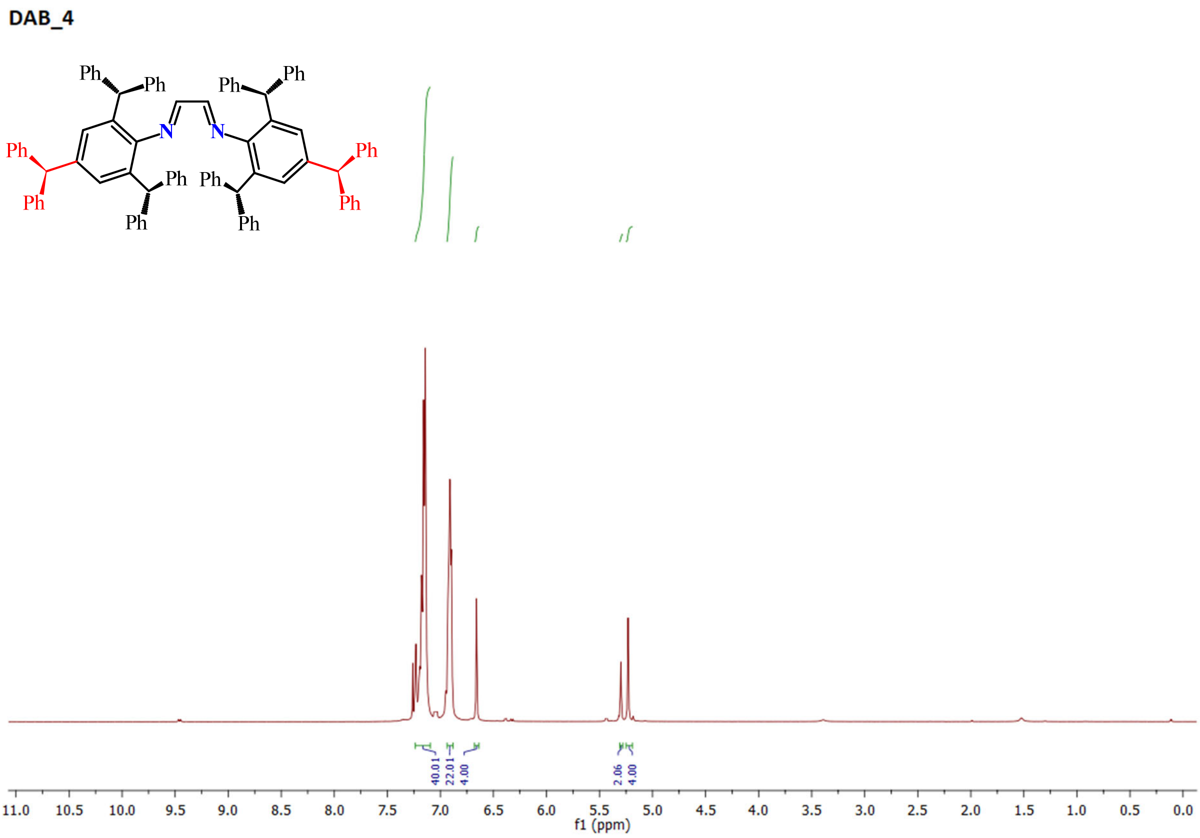

Figure S16. ^{1}H NMR (400 MHz, $CDCl_3$) of **DAB_4**

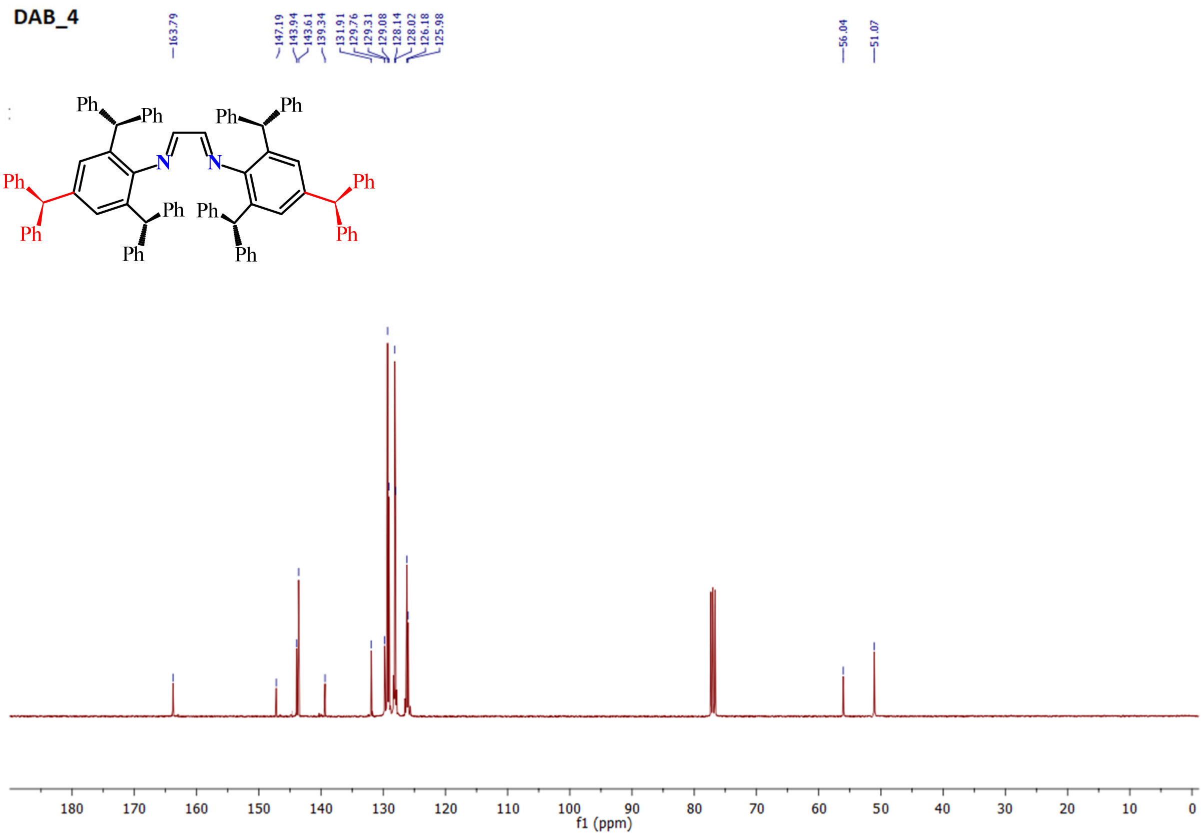

Figure S17. ^{13}C NMR (101 MHz, $CDCl_3$) of **DAB_4**

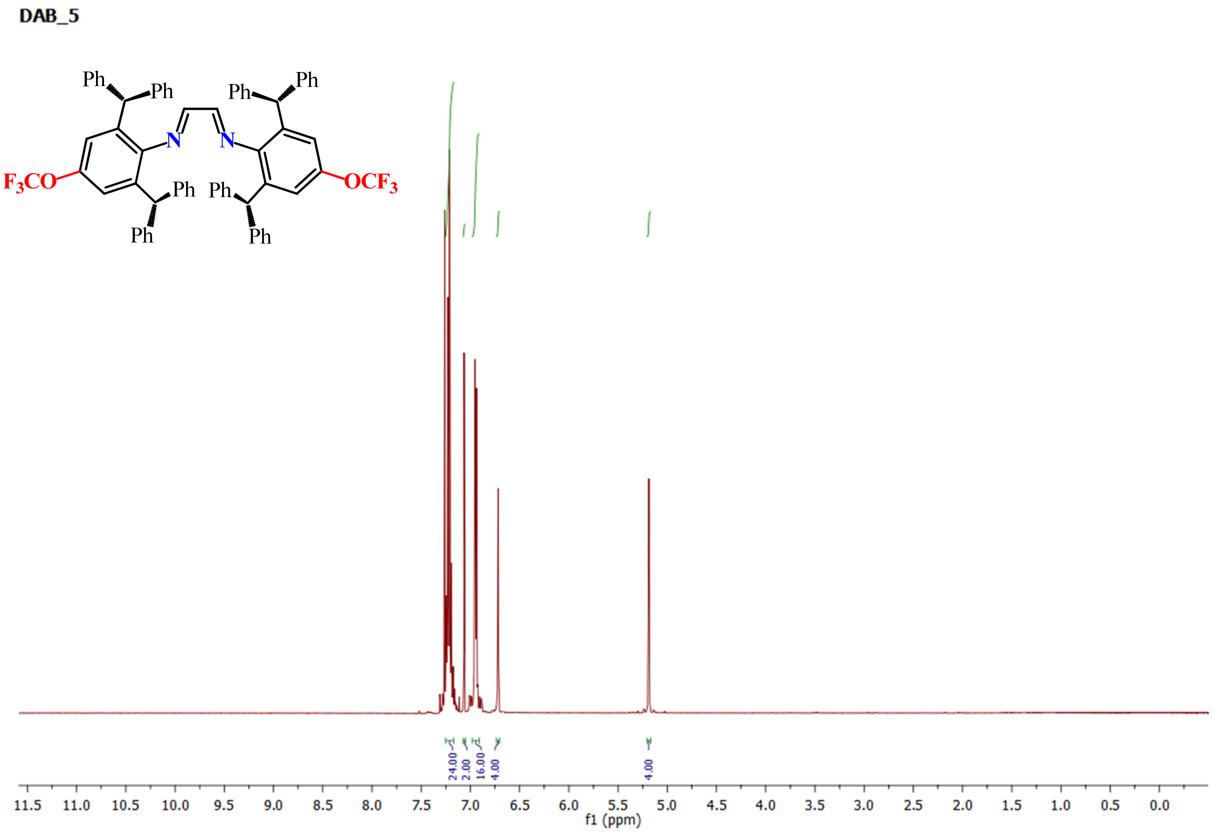

Figure S18. ^{1}H NMR (400 MHz, $CDCl_3$) of **DAB_5**

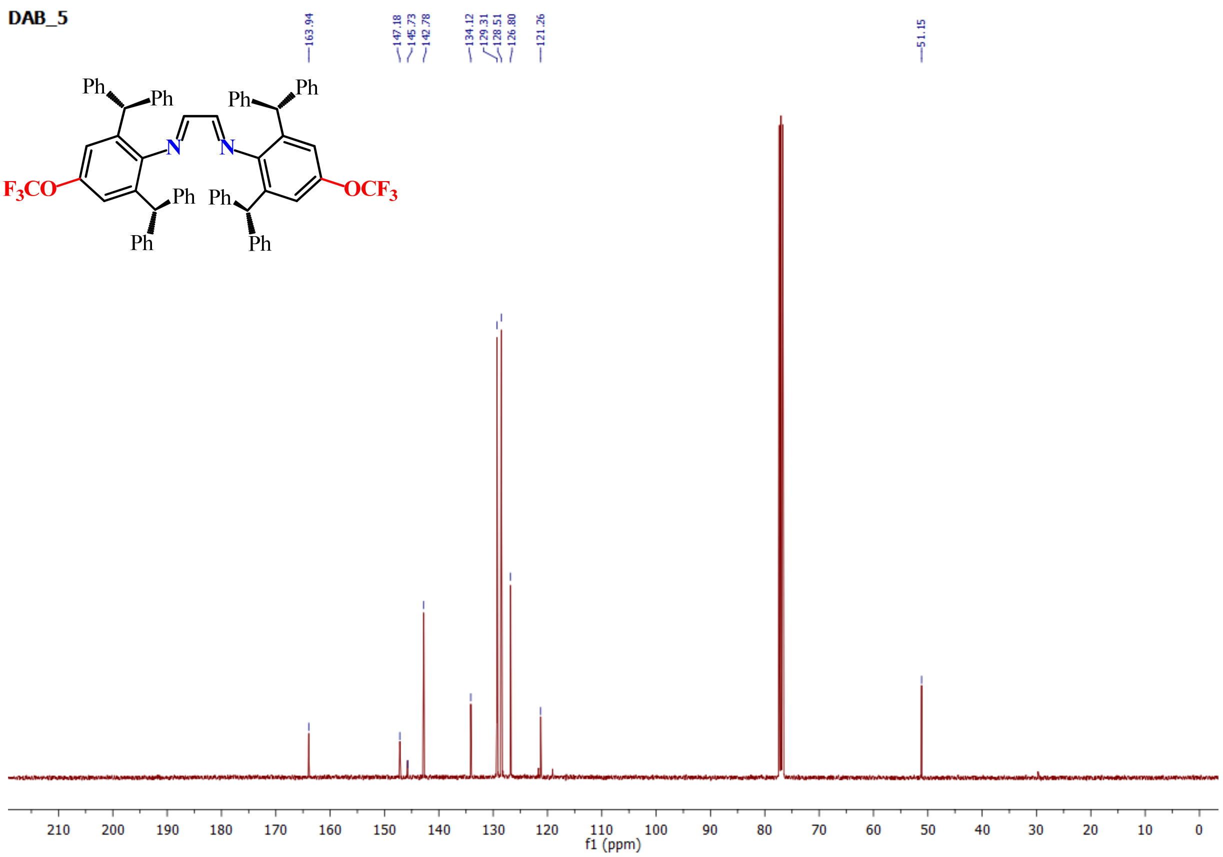

Figure S19. ^{13}C NMR (101 MHz, $CDCl_3$) of **DAB_5**

2.3. NMR spectra of Mn-complexes

Mn1

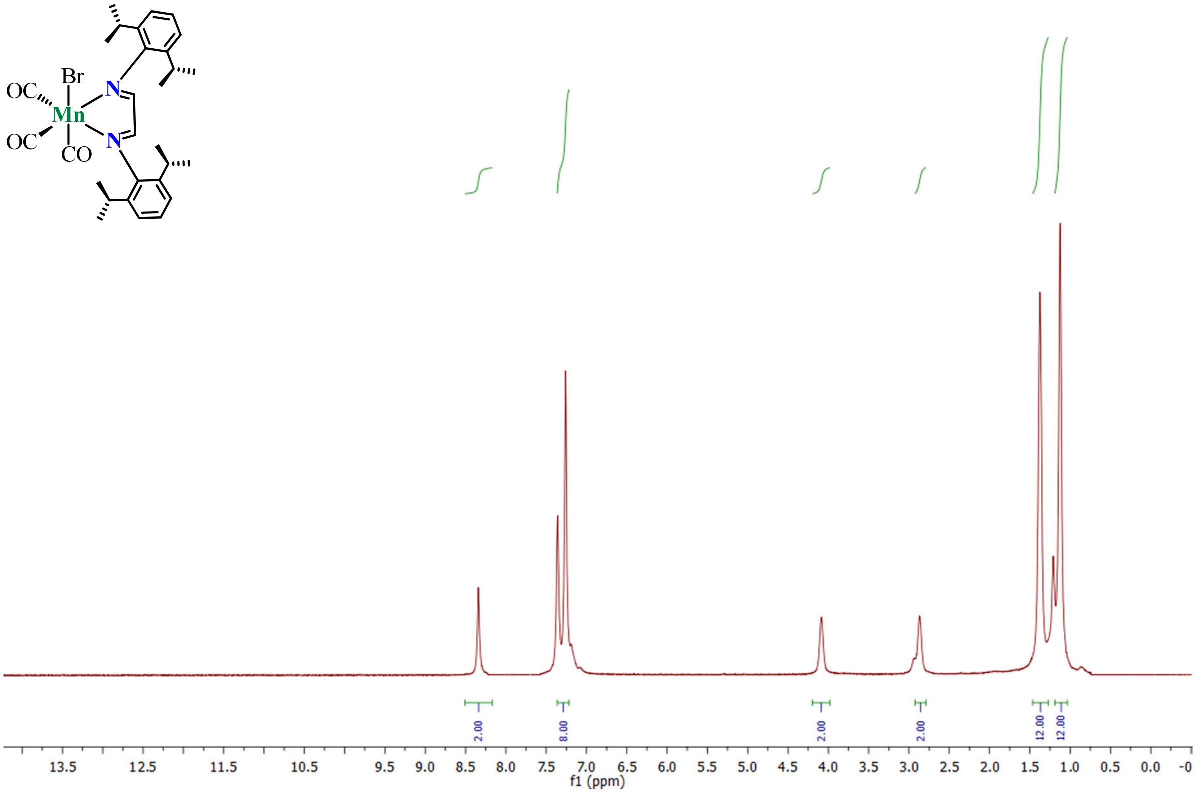

Figure S20. ^{1}H NMR (600 MHz, $CDCl_3$) of **Mn1**

Complex I

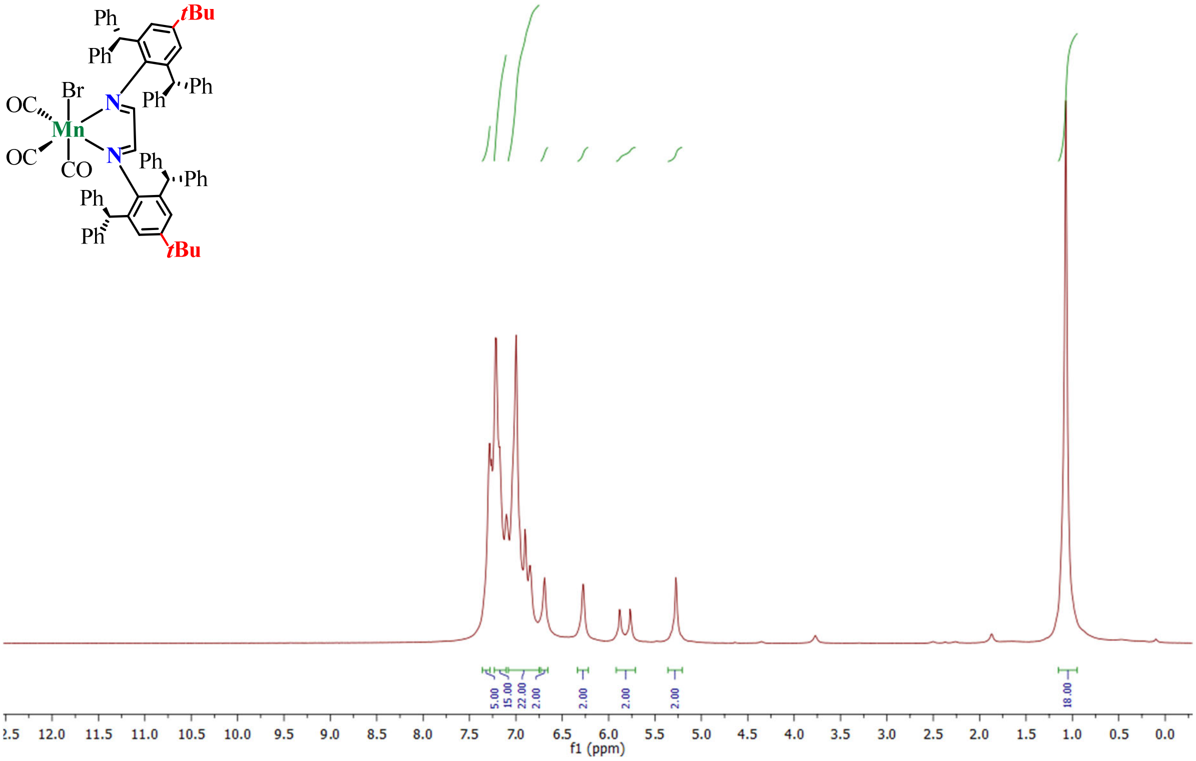

Figure S21. ^{1}H NMR (600 MHz, $CDCl_3$) of complex **I**

Complex II

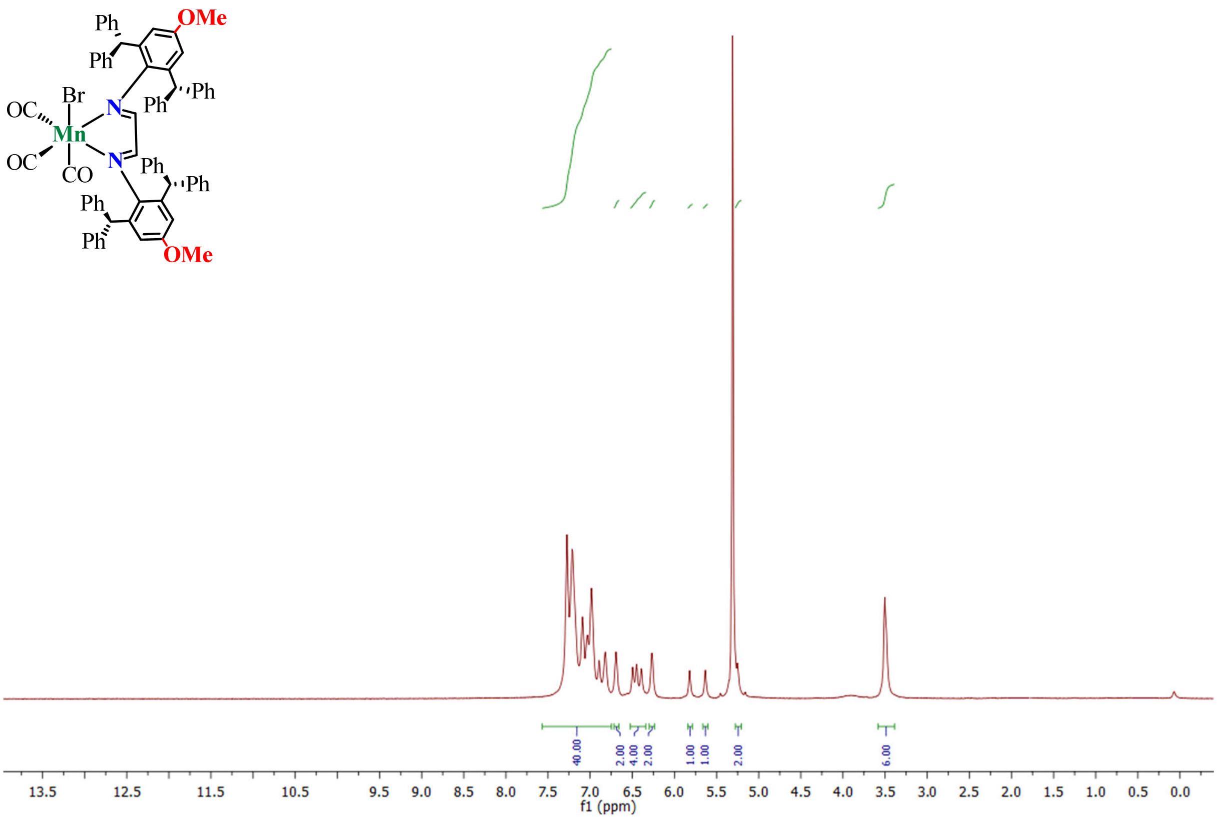

Figure S22. ^{1}H NMR (600 MHz, CD_2Cl_2) of complex **II**

Complex III

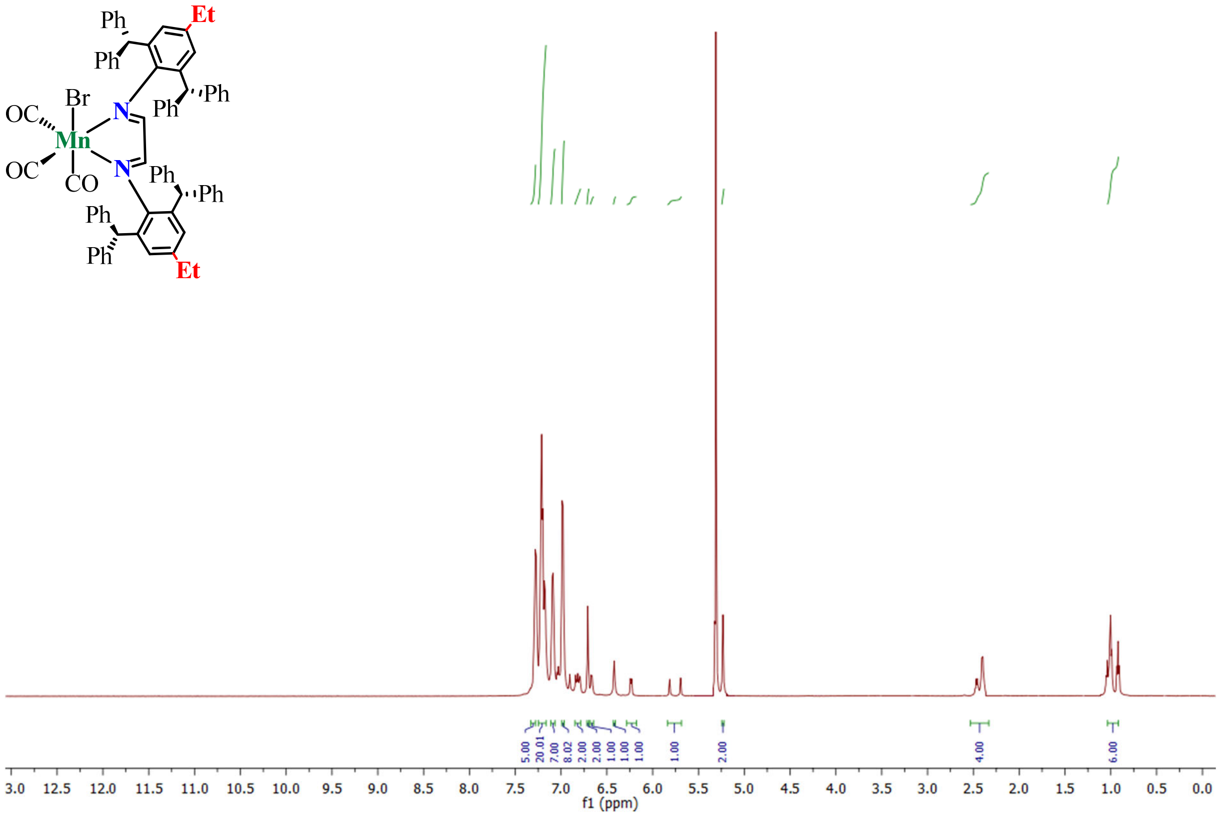

Figure S23. ^{1}H NMR (600 MHz, CD_2Cl_2) of complex **III**

Complex IV

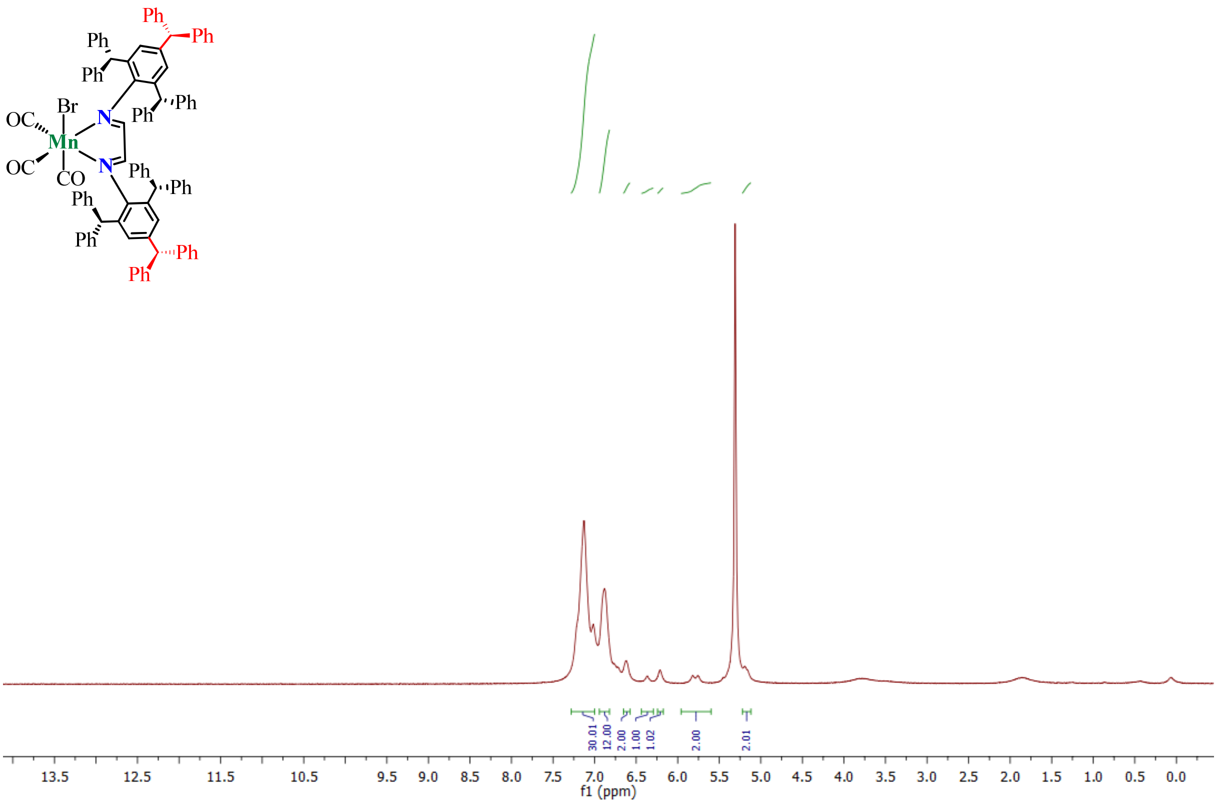

Figure S24. ^{1}H NMR (600 MHz, $CDCl_3$) of complex **IV.**

Complex V

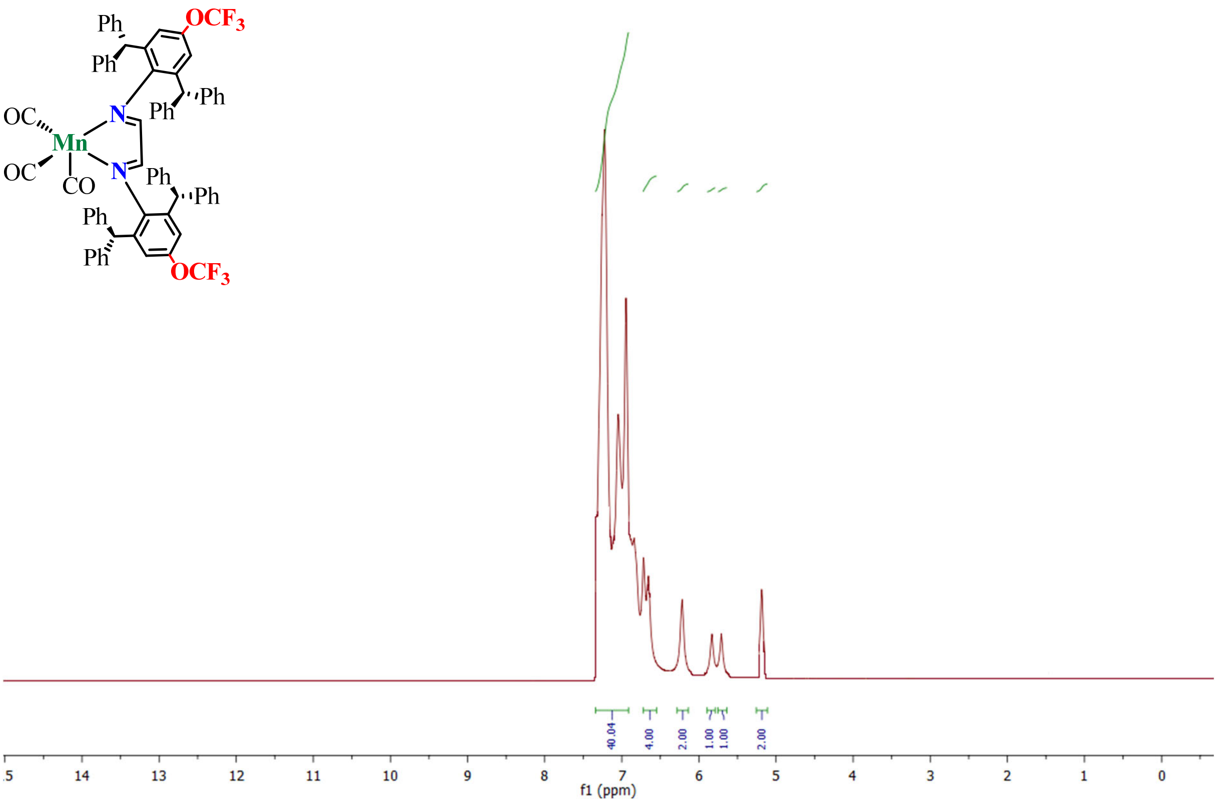

Figure S25. ^{1}H NMR (600 MHz, $CDCl_3$) of complex **V.**

2.4. NMR spectra of representative products

Product P1

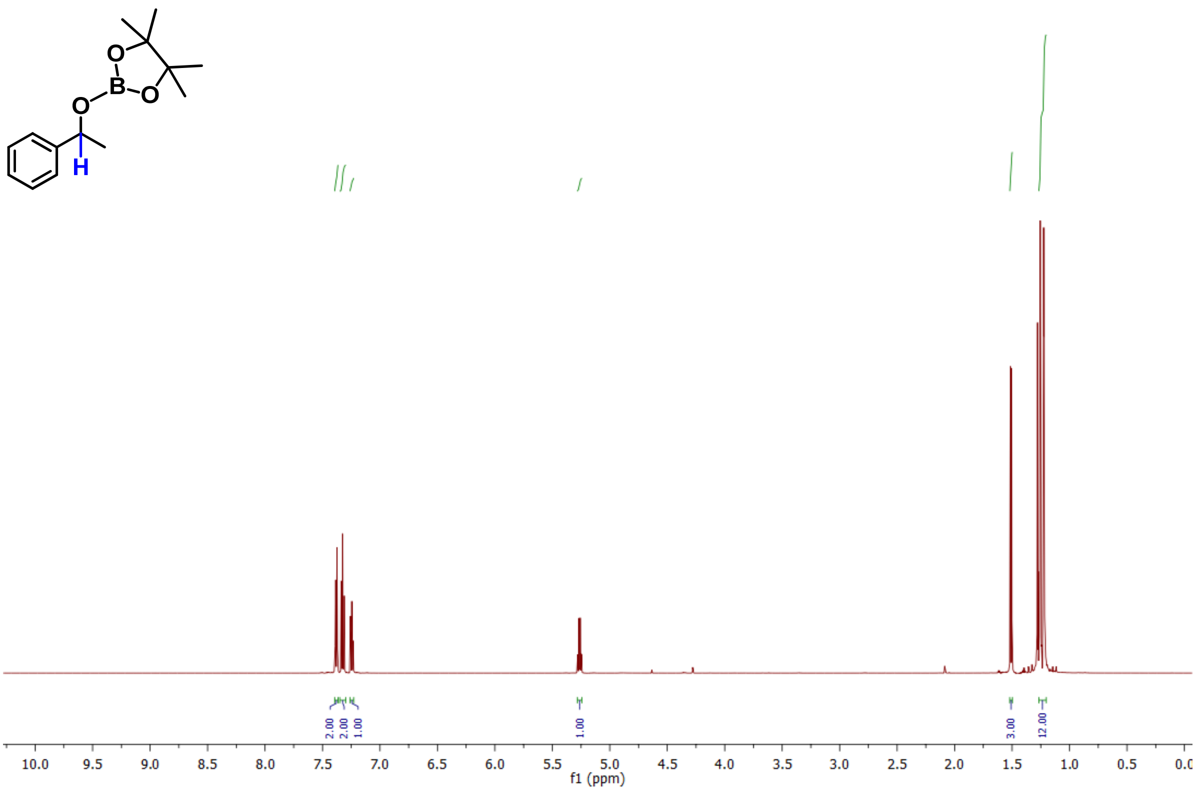

Figure S26. ^{1}H NMR (400 MHz, $CDCl_3$) of product **P1**

Product P1

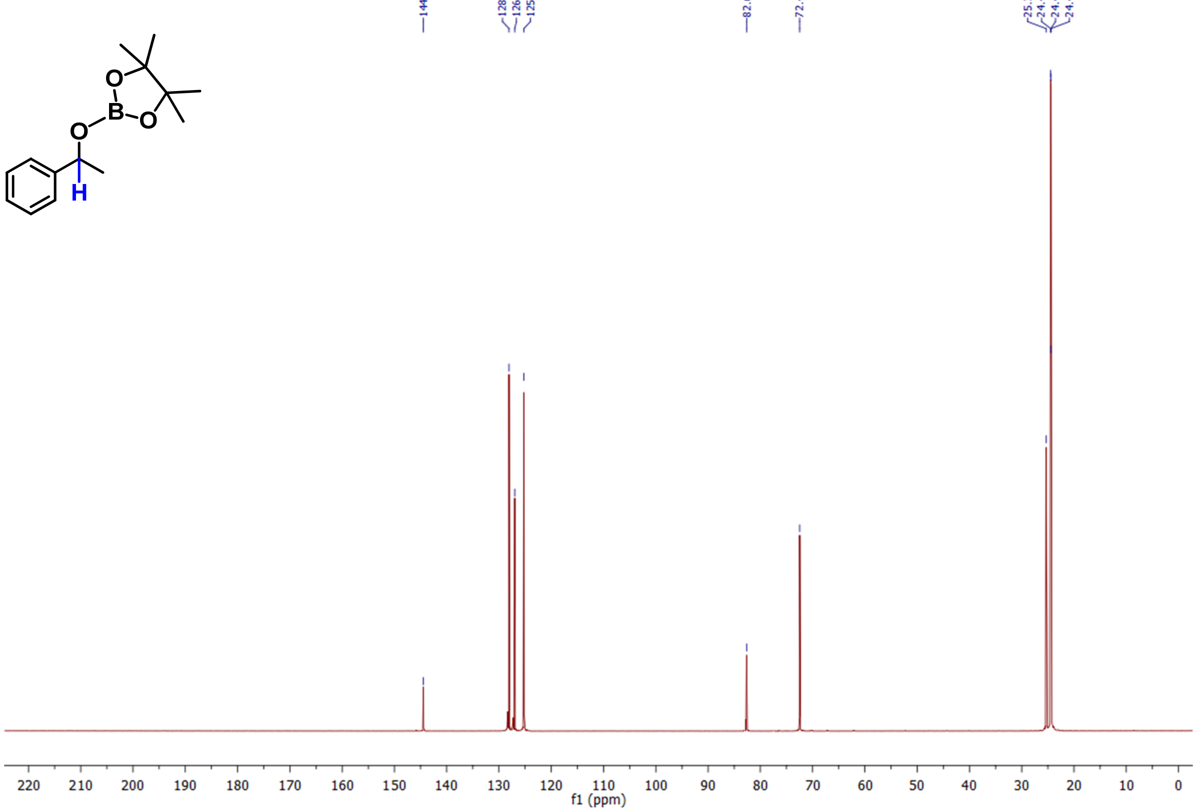

Figure S27. ^{13}C NMR (101 MHz, $CDCl_3$) of product **P1**

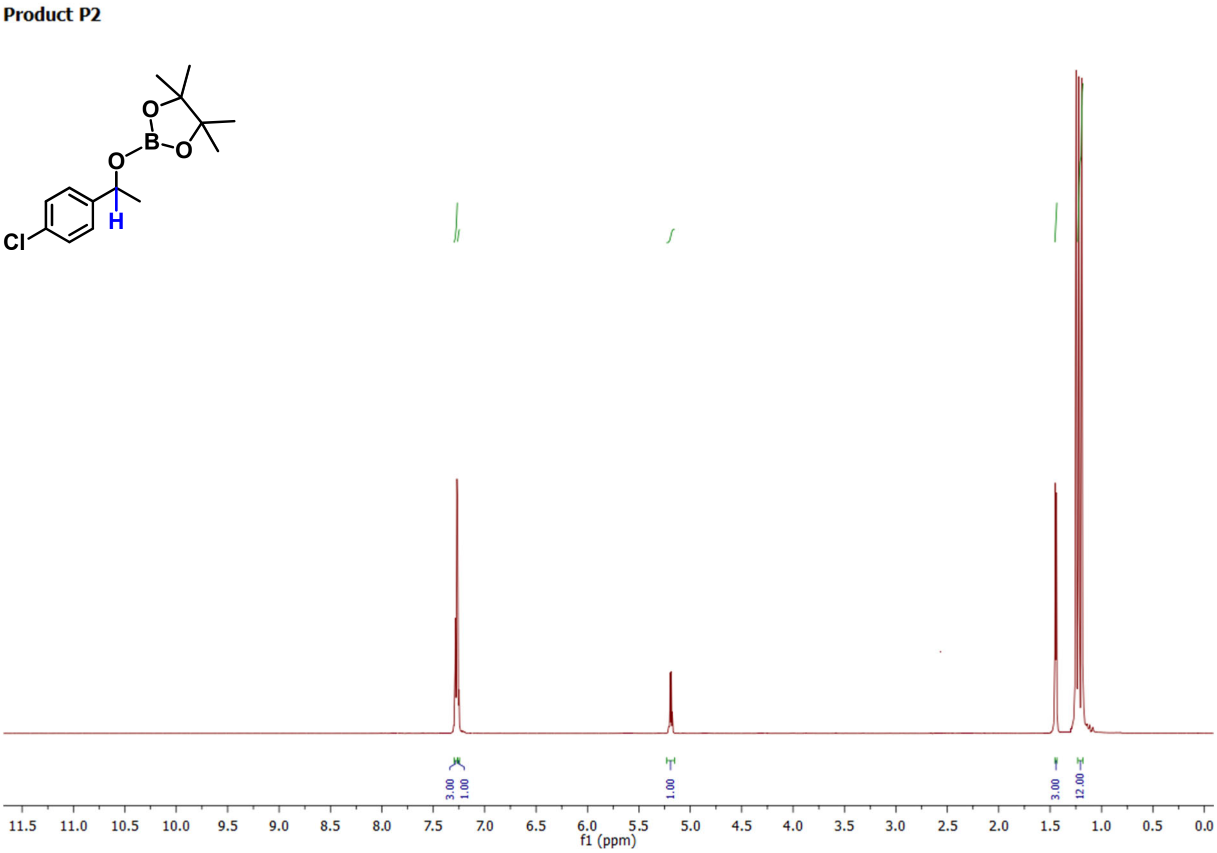

Figure S28. ^{1}H NMR (400 MHz, $CDCl_3$) of product **P2**

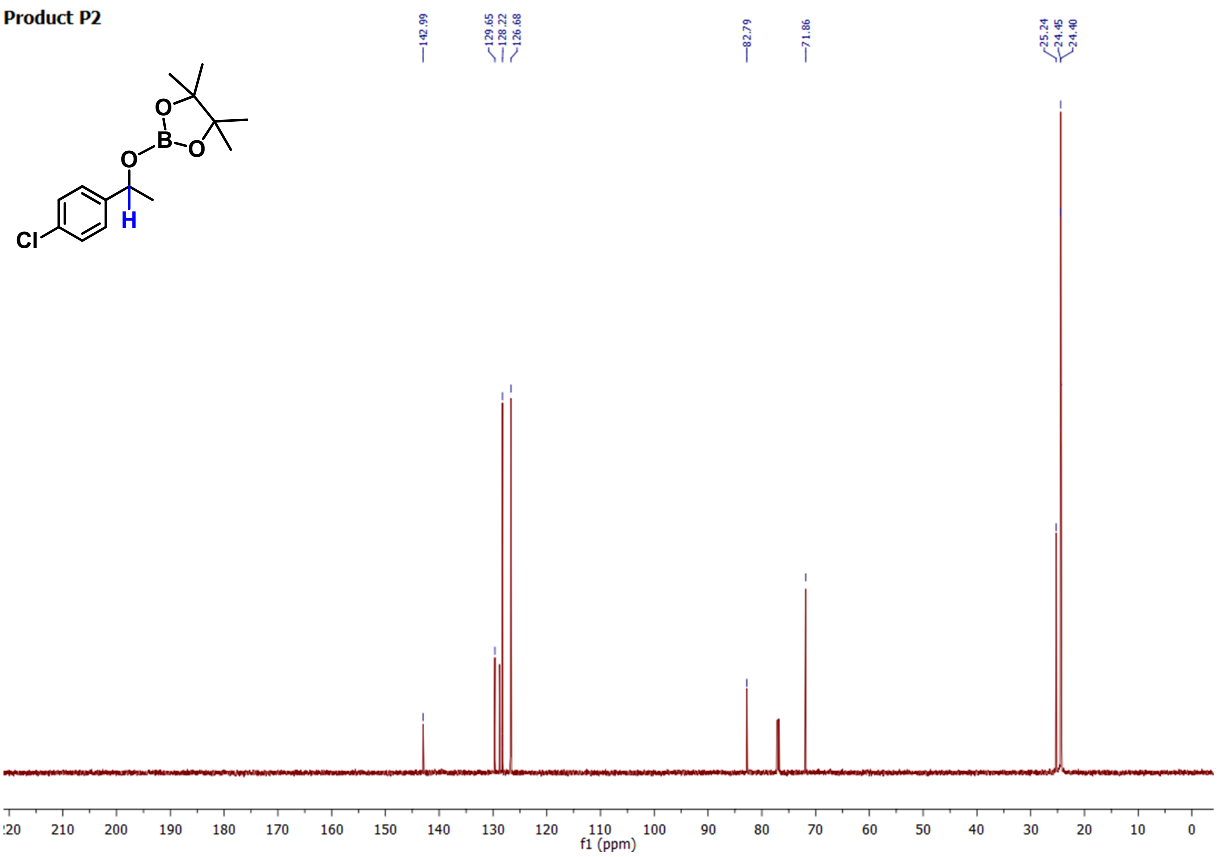

Figure S29. ^{13}C NMR (101 MHz, $CDCl_3$) of product **P2**

Product P8

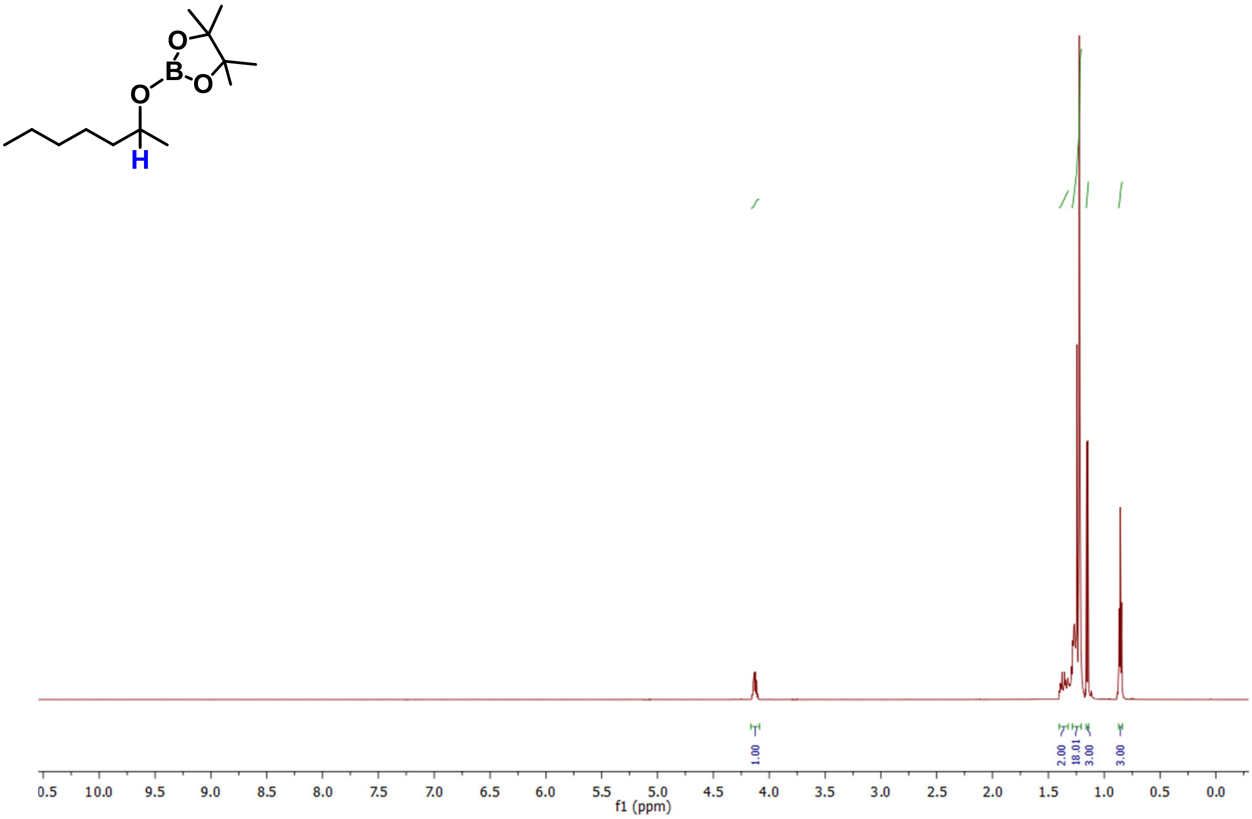

Figure S30. ^{1}H NMR (400 MHz, $CDCl_3$) of product **P8**

Product P8

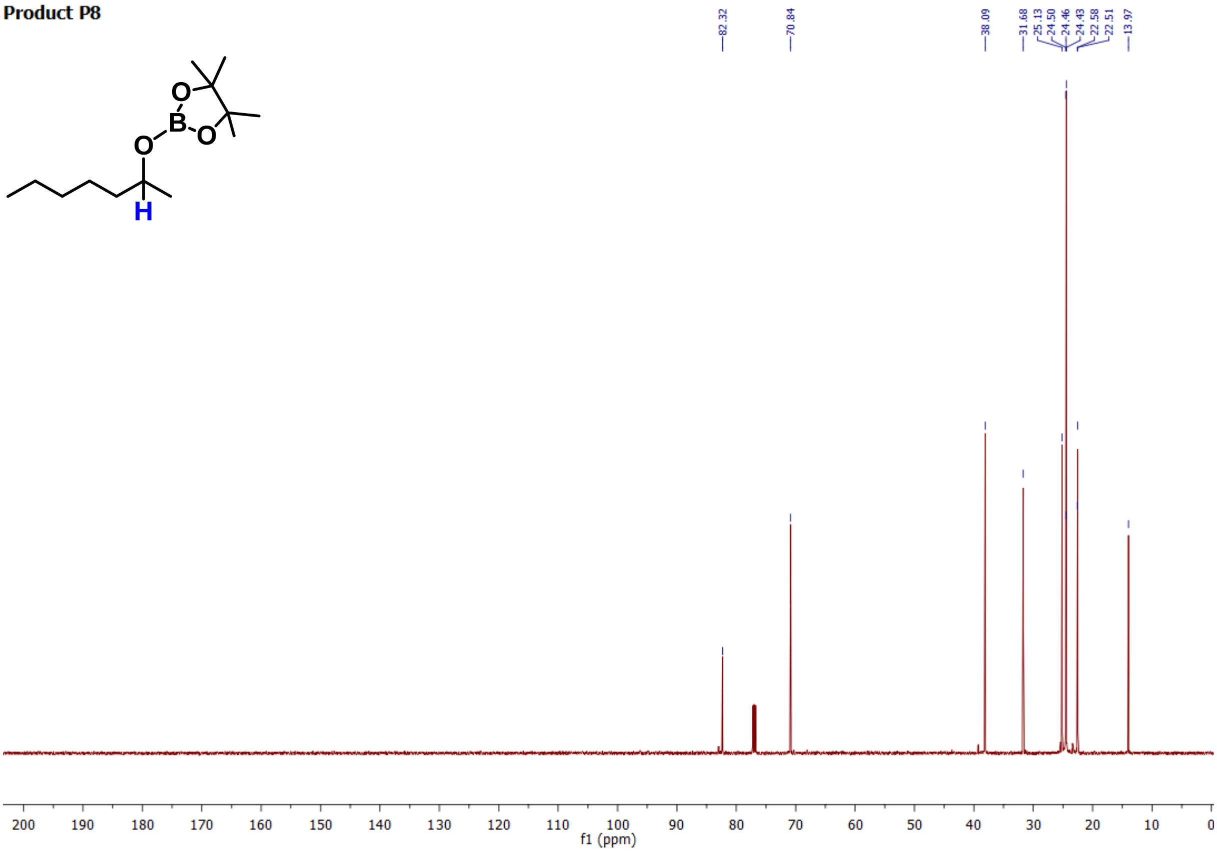

Figure S31. ^{13}C NMR (101 MHz, $CDCl_3$) of product **P8**

Product P1'

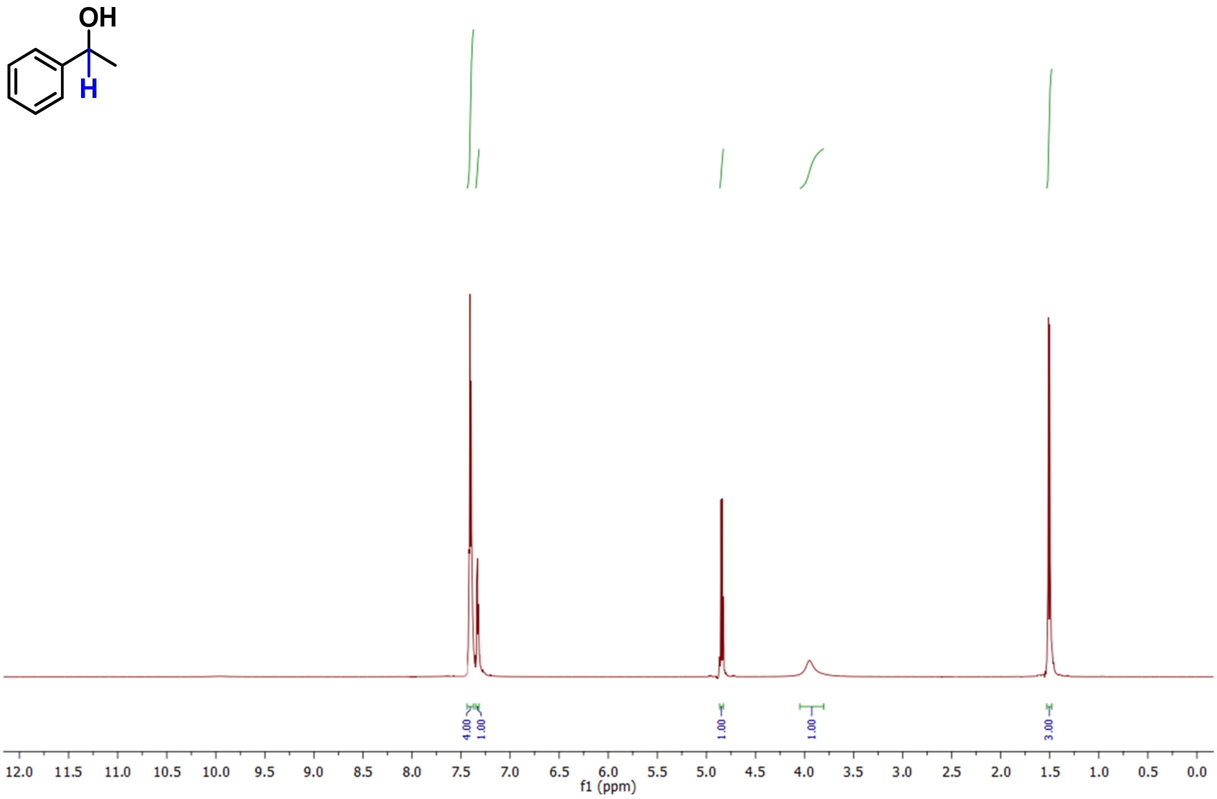

Figure S32. ^{1}H NMR (400 MHz, $CDCl_3$) of product **P1’**

Product P1'

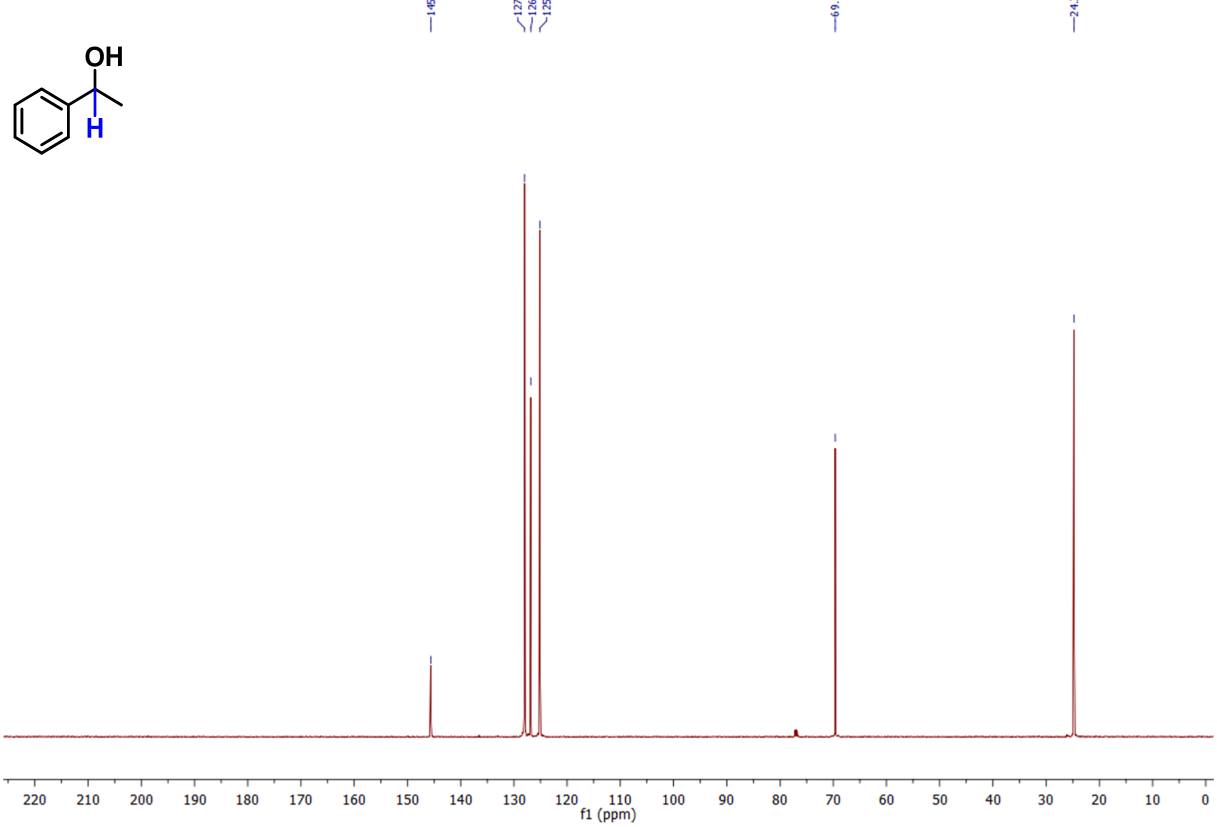

Figure S33. ^{13}C NMR (101 MHz, $CDCl_3$) of product **P1’**

3. XRD analysis

3.1. X-ray crystallography of the A5, DAB_3 and DAB_5

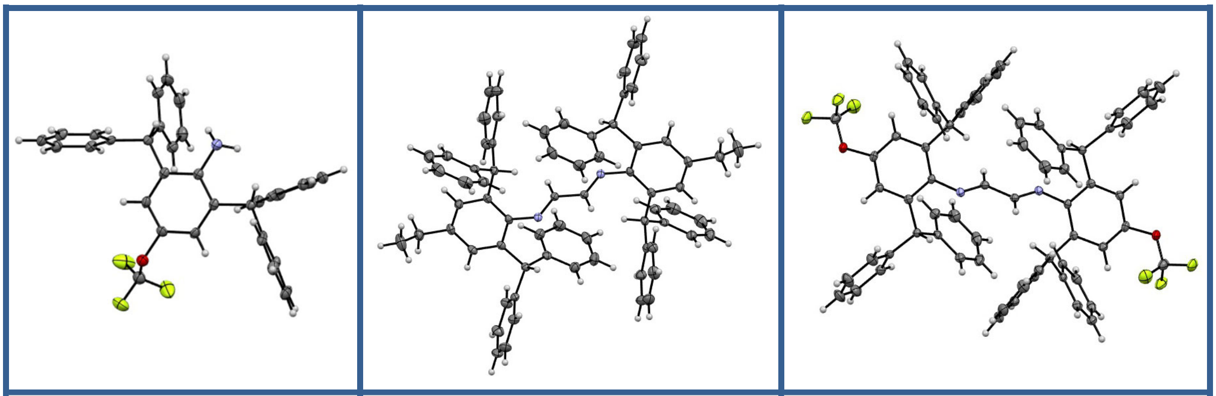

A5	DAB_3	DAB_5

Figure S34. A perspective views of the molecules **A5, DAB_3 and DAB_5.**

Diffraction data were collected by the ω-scan technique on Rigaku XCalibur four-circle diffractometer with EOS CCD detector and graphite-monochromated MoK_{α} radiation (λ=0.71073 Å). The data were corrected for Lorentz-polarization as well as for absorption effects.[S4] Precise unit-cell parameters were determined by a least-squares fit of 1535 (**A5**), 7706 (**DAB_3**), 4860 (**DAB_5**) reflections of the highest intensity, chosen from the whole experiment. The structures were solved with SHELXT-2015[S5] and refined with the full-matrix least-squares procedure on F^2 by SHELXL-2015.[S6] All non-hydrogen atoms were refined anisotropically, hydrogen atoms were placed in idealized positions and refined as 'riding model' with isotropic displacement parameters set at 1.2 (1.5 for CH_3) times Ueq of appropriate carrier atoms.

Table S1 lists the relevant experimental data and refinement details. Crystallographic data for the structural analysis has been deposited with the Cambridge Crystallographic Data Centre, [S7] Nos. CCDC-2430526 (**A5**), CCDC-2474704 (**DAB_3**), CCDC-2430527 (**DAB_5**). Copies of this information may be obtained free of charge from: The Director, CCDC, 12 Union Road, Cambridge, CB2 1EZ, UK. Fax: +44(1223)336-033, e-mail:deposit@ccdc.cam.ac.uk, or www: www.ccdc.cam.ac.uk.

Table S1. Crystal data and structures refinement for the **A5, DAB_3 and DAB_5**

**Compound**	**A5**	**DAB_3**	**DAB_5**
Empirical formula	$C_{33}H_{26}F_3NO$	$C_{74}H_{64}Cl_{12}N_2$	$C_{68}H_{50}F_6N_2O_2$
Formula weight	509.55	1406.67	1041.10
Temperature/K	100	150	100
Crystal system	orthorhombic	triclinic	triclinic
Space group	$Pnma$ (62)	P-1	$P\overline{1}$ (2)
a/Å	17.6353(8)	10.1555(4)	11.1695(7)

b/Å	24.5817(12)	13.9722(4)	11.1784(8)
c/Å	5.8386(3)	14.0184(8)	12.2351(7)
α/°	90	105.917(3)	68.891(6)
β/°	90	110.917(4)	84.664(5)
γ/°	90	99.148(3)	67.904(6)
Volume/Å^3	2531.1(2)	1712.16(14)	1318.87(16)
Z	4	1	1
ρ_{calc}g/cm^3	1.337	1.364	1.311
μ/mm^{-1}	0.095	0.530	0.093
F(000)	1064	726.0	542
Crystal size/mm^3	0.6×0.4×0.3	0.4 × 0.2 × 0.1	0.6×0.2×0.2
Radiation	Mo K_α (λ=0.71073 Å)	Mo Kα (λ = 0.71073)	Mo K_α (λ=0.71073 Å)
2Θ range for data collection/°	6.63 to 56.61 (0.75 Å)	6.504 to 57.844	6.41 to 56.80 (0.75 Å)
Index ranges	-12 ≤ h ≤ 22<br>-24 ≤ k ≤ 29<br>-7 ≤ l ≤ 6	-10 ≤ h ≤ 12,<br>-18 ≤ k ≤ 17,<br>-16 ≤ l ≤ 17	-13 ≤ h ≤ 13<br>-13 ≤ k ≤ 13<br>-14 ≤ l ≤ 16
Reflections collected	6433	16996	15529
Independent reflections	2686<br>R_{int} = 0.0292<br>R_{sigma} = 0.0453	7418<br>R_{int} = 0.0318,<br>R_{sigma} = 0.0504	5587<br>R_{int} = 0.0284<br>R_{sigma} = 0.0370
Data/restraints/parameters	2686/0/185	7418/0/398	5587/0/353
Goodness-of-fit on F^2	1.025	1.047	1.033
Final R indexes [I>=2σ (I)]	R_1 = 0.0511<br>wR_2 = 0.1144	R_1 = 0.0579,<br>wR_2 = 0.1037	R_1 = 0.0433<br>wR_2 = 0.0990
Final R indexes [all data]	R_1 = 0.0756<br>wR_2 = 0.1290	R_1 = 0.0819,<br>wR_2 = 0.1152	R_1 = 0.0591<br>wR_2 = 0.1085
Largest diff. peak/hole / e Å^{-3}	0.90/-0.32	0.73/-0.55	0.43/-0.27

3.2. X-ray crystallography of the complex V

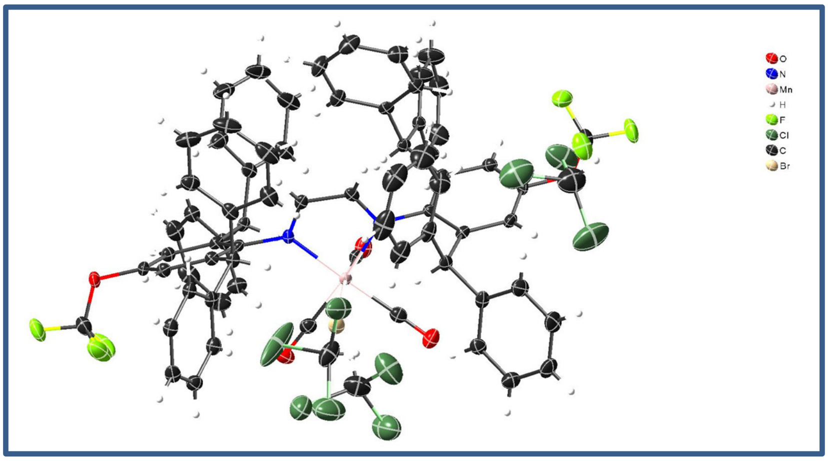

Figure S35. A perspective view of the molecule **V**.

A dark blue, block-shaped crystal was mounted on the goniometer. Data for complex **V** were collected from a single crystal at 100 K on a Bruker D8 QUEST KAPPA diffractometer with a microfocus sealed tube using a multilayer mirror as monochromator and a Bruker PHOTON III CPAD detector. The diffractometer was equipped with an Oxford Cryostream 600 low temperature device and used Mo K_α radiation (λ = 0.71073 Å). All data were integrated with SAINT V8.41, yielding 78736 reflections of which 12814 where independent (average redundancy 6.14) and 90.6% were greater than 2σ(F^2).[S8] A Multi-Scan absorption correction using SADABS 2016/2 was applied.[S9] The structure was solved by dual methods with SHELXT and refined by full-matrix least-squares methods against F^2 using XL.[S5, S6] All non-hydrogen atoms were refined with anisotropic displacement parameters. All hydrogen atoms were refined isotropic on calculated positions using a riding model with their U_{iso} values constrained to 1.2 times the U_{eq} of their pivot atoms.
Table S2 lists the relevant experimental data and refinement details. Crystallographic data for the structural analysis has been deposited with the Cambridge Crystallographic Data Centre, No. CCDC 2488479.[S7] The data can be obtained free of charge from The Cambridge Crystallographic Data Centre via www.ccdc.cam.ac.uk/structures. This report and the CIF file were generated using FinalCif.[S10]

Table S2. Crystal data and structure refinement for the complex **V**

**Compound**	**Complex V**
Empirical formula	$C_{74}H_{53}BrCl_9F_6MnN_2O_5$
Formula weight	1618.13
Temperature/K	100
Crystal system	monoclinic
Space group	$P2_1/c$ (14)
a/Å	14.1232(4)
b/Å	26.2962(8)
c/Å	19.0262(5)
α/°	90
β/°	93.1440(10)
γ/°	90
Volume/Å^3	7055.4(3)
Z	4.0
ρ_{calc}g/cm^3	1.523
μ/mm^{-1}	1.163
F(000)	3272
Crystal size/mm^3	0.1×0.1×0.2
Radiation	dark blue
2Θ range for data collection/°	block
Index ranges	Mo K_α (λ=0.71073 Å)
Reflections collected	3.83 to 50.82 (0.83 Å)

Independent reflections	−17 ≤ h ≤ 17<br>−31 ≤ k ≤ 31<br>−22 ≤ l ≤ 22
Data/restraints/parameters	78736
Goodness-of-fit on F^2	12814<br>R_{int} = 0.0272<br>R_{sigma} = 0.0153
Final R indexes [I>=2σ (I)]	99.4
Final R indexes [all data]	12814 / 0 / 883
Largest diff. peak/hole / e Å^{-3}	1.049

4. References

[S1] V. Yempally, S. J. Kyran, R. K. Raju, W. Y. Fan, E. N. Brothers, D. J. Darensbourg, A. A. Bengali, *Inorg. Chem*., **2014**, *53*, 4081–4088.

[S2] A. Szymańska, M. Nowicki, S. Dea, B. Krupa, J. Szyling, J. Walkowiak, *Adv. Synth. Catal*., **2025**, 7354590.

[S3] D. Willcox, J. L. Carden, A. J. Ruddy, P. D. Newman, R. L. Melen, *Dalton Trans*, **2020**, *49*, 2417-2420.

[S4] CrysAlisPro version 1.171.44.108a (Rigaku OD, **2025**).

[S5] G. M. Sheldrick, *Acta Crystallogr*., **2015**, 71, 3-8.

[S6] G. M. Sheldrick, *Acta Crystallogr*., **2015**, 71, 3-8.

[S7] C. R. Groom, I. J. Bruno, M. P. Lightfoot, S. C. Ward, *Acta Cryst*., **2016**, B72, 171–179.

[S8] Bruker, SAINT, V8.41, Bruker AXS Inc., Madison, Wisconsin, USA.

[S9] L. Krause, R. Herbst-Irmer, G. M. Sheldrick, D. Stalke, *J. Appl. Cryst*., **2015**, *48*, 3–10.

[S10] D. Kratzert, *FinalCif*, *(Bruker Edition)*, https://dkratzert.de/finalcif.html.
